# Integrating deep convolutional surrogate solvers and particle swarm optimization for efficient inverse design of plasmonic patch nanoantennas

**DOI:** 10.1515/nanoph-2024-0195

**Published:** 2024-08-02

**Authors:** Saeed Hemayat, Sina Moayed Baharlou, Alexander Sergienko, Abdoulaye Ndao

**Affiliations:** Department of Electrical and Computer Engineering, University of California San Diego, La Jolla, CA 92093, USA; Department of Electrical and Computer Engineering and Photonics Center, 8784 Boston University, 8 Saint Mary’s Street, Boston, MA 02215, USA

**Keywords:** deep learning, inverse design, plasmonics, nanoantennas

## Abstract

Plasmonic nanoantennas with suitable far-field characteristics are of huge interest for utilization in optical wireless links, inter-/intrachip communications, LiDARs, and photonic integrated circuits due to their exceptional modal confinement. Despite its success in shaping robust antenna design theories in radio frequency and millimeter-wave regimes, conventional transmission line theory finds its validity diminished in the optical frequencies, leading to a noticeable void in a generalized theory for antenna design in the optical domain. By utilizing neural networks, and through a one-time training of the network, one can transform the plasmonic nanoantennas design into an automated, data-driven task. In this work, we have developed a multi-head deep convolutional neural network serving as an efficient inverse-design framework for plasmonic patch nanoantennas. Our framework is designed with the main goal of determining the optimal geometries of nanoantennas to achieve the desired (inquired by the designer) *S*_11_ and radiation pattern simultaneously. The proposed approach preserves the one-to-many mappings, enabling us to generate diverse designs. In addition, apart from the primary fabrication limitations that were considered while generating the dataset, further design and fabrication constraints can also be applied after the training process. In addition to possessing an exceptionally rapid surrogate solver capable of predicting *S*_11_ and radiation patterns throughout the entire design frequency spectrum, we are introducing what we believe to be the pioneering inverse design network. This network enables the creation of efficient plasmonic antennas while concurrently accommodating customizable queries for both *S*_11_ and radiation patterns, achieving remarkable accuracy within a single network framework. Our framework is capable of designing a wide range of devices, including single band, dual band, and broadband antennas, with directivities and radiation efficiencies reaching 11.07 dBi and 75 %, respectively, for a single patch. The proposed approach has been developed as a transformative shift in the inverse design of photonics components, with its impact extending beyond antenna design, opening a new paradigm toward real-time design of application-specific nanophotonic devices.

## 1 Introduction

Control and manipulation of light at the nanoscale is considered as one of the cornerstones of modern optics, with the potential to revolutionize scientific and technological advances. Through the years, light manipulation has been implemented through various approaches such as photonic crystals [1], [2], metamaterials [3], [4], metasurfaces [5]–[15], and plasmonic structures due to their unprecedented ability to locally control and manipulate the incident light at the nanoscale [16]–[20]. Plasmonic nanoantennas serve a crucial role in a wide range of applications such as plasmonic lenses [21], [22], plasmonic tweezers [23]–[25], intra-/interchip optical communications [26], [27], LiDARs [28], augmented reality and holography [29], [30], imaging [31], and in surface-enhanced Raman spectroscopy (SERS) [32] due to their unique ability to guide and confine light at the nanoscale. To date, plasmonic nanoantennas are mainly used for near-field applications and lack a robust far-field performance and a generalized far-field design methodology. Although conventional antenna theory has been successful in shaping the design theory and techniques in low-frequency regimes such as radio frequency (RF) or mm-wave, as we venture toward the optical domain, due to the radically different wave-matter interactions, the validity of this theory diminishes significantly [33]. By leveraging neural networks, we can convert this problem into an automated, data-driven task.

Data-driven methods such as deep neural networks (DNNs) are receiving significant attention owing to their remarkable success in computer vision [34], [35], natural language processing [36], [37], and speech recognition [38]. In nanophotonics, DNNs have been used to replace the complex and time-consuming design procedures by approximating the electromagnetic simulations and learning the inverse process [39]–[51], predicting the fabrication imperfections [52], and postfabrication appearance [53]. While promising, DNNs face challenges with inverse problems due to their reliance on a large number of labeled samples (i.e., devices with simulated responses), which grow exponentially with additional degrees of freedom of the device. Also, discriminative neural networks may lead to suboptimal results due to the existing nonuniqueness in inverse problems. Prior studies addressed the inverse design problem using discriminative networks in combination with brute forcing [54], analytical gradient [41], and evolutionary algorithms [55]–[57]. Tandem networks have been utilized in various works [58], [59], and generative models such as variational autoencoders [60], [61] and generative adversarial networks [43], [62]–[64] have been adapted to enhance the design with more degrees of freedom. However, these methods face severe constraints, such as inadvertent discarding of desirable devices in tandem models due to transforming the one-to-many mappings to one-to-one mappings, challenges in encapsulating fabrication constraints, and difficulties in training generative models that may lead to blurry and inaccurate results [65]. In addition, the generative models suffer from mode collapses, limiting their ability to generate multiple diverse results.

In this work, we have developed an inverse-design framework for efficiently designing plasmonic patch nanoantennas to overcome the aforementioned obstacles. Our framework is capable of determining the optimal configuration of nanoantennas to achieve the desired and physically possible *S*_11_ and radiation pattern. The proposed framework is developed based on the pseudo-inverse function. It utilizes a multi-head deep convolutional neural network as a surrogate solver to accurately estimate the *S*_11_ and the radiation pattern of a given device across the entire frequency range. This is orders of magnitude faster than numerical simulations. The particle swarm optimization (PSO) is used in conjunction with the surrogate solver to efficiently search the design space and locate the desired devices. Following the search, a clustering algorithm is applied to identify multiple diverse results. Contrary to most NN-based inverse-design methods, our proposed approach preserves the one-to-many mappings. It allows the designer to choose from multiple diverse devices for a given design problem. The framework enables the designer to add fabrication constraints even after the training process and generate the desired devices through complex queries, enhancing customization in the design process. To the best of our knowledge, this is the first time that a neural network-based inverse design framework encapsulates all of the mentioned properties while maintaining simplicity and fast runtimes. The proposed framework can design a wide range of devices with the desired characteristics, including single band, dual band, and broadband antennas with a maximum directivity of up to 11.07 dBi and radiation efficiencies reaching almost 75 % for a single patch. The proposed approach has been developed to serve as a transformative foundation in inverse design, with its impact extending beyond antenna design and toward real-time design of application-specific nanophotonic devices.

## 2 Deep learning-based inverse design framework

The conventional design process starts with a structure or a set of known input parameters and obtains the corresponding outcome afterward. However, in the inverse design, the process works the other way around. The designer starts with a set of known desired outputs, and the goal is to discover the structure or parameters that can produce those specific outcomes. This process is of great importance because automating it with artificial intelligence (AI) and data-driven methods can significantly accelerate the design process and save time and resources. Additionally, it is possible to identify new and previously unattainable devices that outperform the existing solutions.

**Figure 1:**
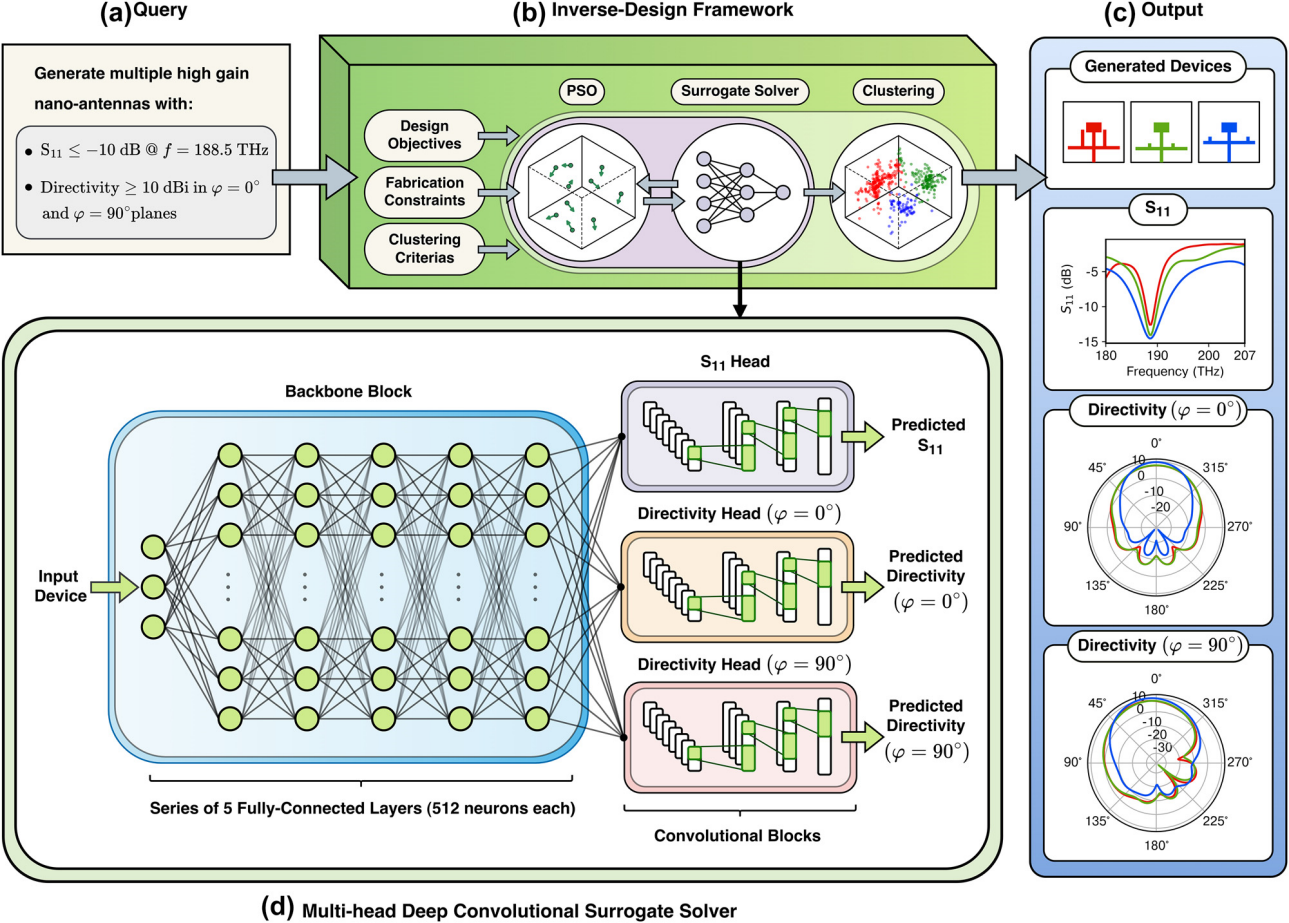
An overview of the proposed inverse-design framework. Our approach is based on solving the pseudo-inverse function. It employs a deep neural network as a surrogate solver to replace the computationally expensive and time-consuming simulation process and uses an optimization algorithm to search the design space for the desired devices. Our approach preserves the one-to-many mappings, allowing the generation of multiple devices and accommodating fabrication constraints. The proposed framework operates by taking a requested response in the form of a query and a set of fabrication constraints as input. An objective function is then defined based on these inputs, which must be minimized using the PSO. A multi-head deep convolutional neural network is trained to perform as a surrogate solver and quickly predict the device responses, including the device *S*_11_ and radiation patterns. The PSO efficiently explores the design space using the surrogate solver and identifies the desired devices. Finally, a clustering algorithm is applied to generate a diverse set of multiple devices. (a) The desired responses in the form of a query, (b) our inverse design framework, (c) generated devices given the desired response, and (d) multi-head deep convolutional surrogate solver.

Our inverse design framework utilizes a deep neural network (serving as a surrogate solver) to model the simulation process and uses PSO to search the design space. The surrogate solver replaces the computationally intensive numerical simulation process, enabling the PSO algorithm to explore the design space efficiently and identify the devices with desired responses. The proposed framework comprises three components: the “Multi-head Convolutional Surrogate Solver,” the “Particle Swarm Optimization Algorithm,” and the “Clustering Algorithm” (as shown in Figure 1(b)). Together, these components generate a collection of feasible and manufacturable nanoantennas with desired responses. The framework takes a desired response in a form of a query (Figure 1(a)) and generates a set of devices that exhibit those responses (Figure 1(c)). The query consists of a set of high-level conditions the desired device must meet. Each condition is represented as a cost function that the PSO aims to minimize (named design objectives). In addition to the query, the designer can define fabrication constraints and clustering parameters to specify the extraction of multiple devices. Fabrication constraints can be incorporated into the inverse process by either adding them as an extra cost function to the PSO objective function or by limiting the search space of the PSO algorithm. After defining the search space and the objective function, PSO begins optimizing the objective function: in other words, PSO will search the device space for a set of devices that meets the requirements. In the search process, several devices need to be evaluated (i.e., their responses should be simulated). This evaluation is done using the multi-head deep convolutional surrogate solver, which is trained to approximate the responses of the given device. The architectural detail of the surrogate solver is illustrated in Figure 1(d). The surrogate solver enables the PSO to search the device space in a matter of seconds. PSO iteratively collaborates with the surrogate solver until convergence. After the device space is searched, the mean shift algorithm clusters the resulting particles to locate a set of admissible solutions, instead of just one. It then outputs the set of devices, as illustrated in Figure 1(c). The surrogate solver and the inverse process are explained in detail in Sections 2.3 and 2.4, respectively.

We refer to our approach as solving the pseudo-inverse function, which involves modeling the forward process using the neural network and optimizing it to find the desired devices. In the following, we will explain the pseudo-inverse function, its advantages, and why modeling the inverse process directly using neural networks has challenges and would not provide the mentioned benefits.

Assuming that the parameters and response of the nanoantenna can be represented as vectors $d\in R^{n}$ and $r\in R^{m}$ , where *n* and *m* are the dimensions of the device and response space, respectively. We will define the function *f* : *d* → *r* as the forward design function. This function is known and well-defined, meaning that for any input device *d*, a single response *r* is produced. This function can be evaluated through time-consuming numerical simulations.

Considering the above notations, the function *f* ^−1^ : *r* → *d* is called the inverse design function. The ability to evaluate, learn, and estimate this function plays a crucial role in inverse design tasks because a target device with a set of desired responses can be determined by evaluating this function. However, *f* is neither injective nor surjective, leading to the fact that it does not have a well-defined inverse function *f* ^−1^. As a result, some responses cannot be generated by any device, while multiple devices can generate others (see Supplementary Figure S1), resulting in one-to-many mappings. When using machine learning models such as discriminative neural networks to model the inverse function, one of the main challenges is dealing with one-to-many mappings in the dataset. This is because discriminative neural networks are designed to learn one-to-one mappings, and modeling the inverse function using these networks would result in poor convergence and inaccurate results. A workaround to overcome this issue is converting *d* → *r* to a bijective mapping [58], resulting in a one-to-one inverse function that removes potentially valuable devices from the device space.

Contrary to methods that do not preserve the one-to-many mappings, our inverse design framework is based on solving the pseudo-inverse function, denoted as *f* ^†^, with the property of $f\left(f^{†}\left(r\right)\right)\approx r$ , which can preserve the one-to-many mappings. This function is defined as $f^{†}\left(r\right)=D$ , where *D* is a set of possible solutions (devices), each satisfying the following condition:

(1) $$D=\left\{d\in R^{n},{\left\|f\left(d\right)-r\right\|}^{2}<\varepsilon \right\},$$

where *ɛ* is a predefined threshold value indicating the maximum allowed discrepancy between the desired and the target responses. A set of possible solutions *D* can be obtained by a search or an optimization algorithm. This process includes multiple evaluations of the forward function (the numerical simulations), which can be time-consuming. To speed up this process, we have developed and trained a multi-head convolutional neural network as the surrogate solver to approximate the simulation function, allowing for a fast and parallel evaluation of *f*. Additionally, we have injected the designer’s knowledge by incorporating a predefined structure and considering a set of prior fabrication constraints (such as minimum feature sizes and minimum distances between the T-stubs with the feed and the patch), reducing the device space significantly. As a result, our pseudo-inverse function can be defined as follows:

(2) $$D=\left\{d\in R^{k},{\left\|\hat{f}\left(d\right)-r\right\|}^{2}<\varepsilon \right\},$$

where *k* is the dimension of the reduced device space *k* ≪ *n*, and $\hat{f}\left(d\right)$ is the approximated simulation function using the surrogate solver. The values of *d* are also limited to the range of motion of the parameters of a predefined structure. Having a fast surrogate solver and a bounded device space, PSO is utilized consequently for efficient exploration of the device space and determining possible solutions *D*. Since the one-to-many mappings remain intact, multiple diverse solutions can be determined by identifying the clusters formed by the PSO algorithm using a clustering algorithm and obtaining the local minima in each cluster. Notably, the proposed surrogate solver performs the PSO’s particle evaluation stages in parallel, allowing all particles to be evaluated simultaneously, which hugely increases the efficiency of the inverse algorithm. For instance, designing a single device using our framework requires an average of 20 iterations of the PSO, with 512 devices evaluated in each iteration. The average execution time of our inverse design process is 0.08 ± 0.02 s per device, depending on the hardware. It is noteworthy that, without the surrogate solver, the execution time to design one device would take more than 56 h.

One of the most important advantages of the proposed approach lies in the fact that fabrication constraints can be imposed by either limiting the search space (e.g., fixing a parameter, reducing the range of a variable) or penalizing the regions where the fabrication constraints are not met, after the training and learning process (one does not need to limit the training dataset space to a dataset where fabrication constraints have already been applied, which will eliminate many of the potential devices). Furthermore, the designer can choose a less sensitive device to fabrication imperfections, as the one-to-many mapping is preserved and multiple solutions are discovered.

### 2.1 Dataset

Deep neural networks often require large-scale datasets for accurate predictions. The number of training samples varies based on different factors, including the input and output dimensions and the mapping complexity. Gathering a large-scale dataset is both time-consuming and costly, especially when training a physical surrogate solver, as the generated devices must also go through the simulation process.

In this work, by exploiting the designer’s knowledge, we have significantly reduced the dimensions of the device space and have bound it to meet fabrication limitations, such as the overall size of the device, minimum feature sizes, minimum distance between two elements, and compatibility of the device with current fabrication technologies. Our devices have three or four degrees of freedom, and we are specifically interested in the device’s *S*_11_ response and radiation pattern. The *S*_11_ frequency range spans from 180 THz to 207 THz, and we have sampled the data at 96 points within this range. Additionally, we have captured the directivity of the device at four frequencies of interest (185 THz, 188.5 THz, 193.5 THz, 198.5 THz). For each frequency, we have two cuts of the radiation pattern in *φ* = 0° and *φ* = 90°, and these data have been sampled through 72 points. This makes our device space belong to $d\in R^{3}$ or $d\in R^{4}$ , and our response space belongs to $r\in R^{672}$ . In the text, the responses are denoted individually in the form of $r_{s_{11}}\in R^{96},r_{\varphi =0{}^{\circ}}\in R^{4\times 72},r_{\varphi =90{}^{\circ}}\in R^{4\times 72}$ , or in a concatenated and flattened form of $r={\left[r_{s_{11}}^{⊤},r_{\varphi =0{}^{\circ}}^{⊤},r_{\varphi =90{}^{\circ}}^{⊤}\right]}^{⊤}$ .

A dataset of 50,000 samples has been generated and simulated with their corresponding responses for the device with three degrees of freedom using the CST Studio. Throughout this process, Latin hypercube sampling (LHS) [66] has been used to generate random samples due to its efficiency in covering the parameter space compared to simple random sampling (see Supplementary Section S1.1 for the importance of using LHS). About 90 % of the generated data has been used for training (45,000 samples), and the remaining 10 % has been kept for validation and testing (2,500 each). The validation set evaluates the surrogate model to obtain the optimal architecture and training hyperparameters. In contrast, the test set determines the model’s final accuracy. We have also verified that all the generated samples are unique, with no leakage of validation or test sets.

Throughout the text, the experiments and the results are reported for the dataset with three degrees of freedom, and the quantitative and qualitative results for the device with four degrees of freedom can be found in the Supplementary Material.

Upon further analysis of the gathered dataset, the presence of one-to-many mappings was confirmed. This was achieved by extracting distinct devices from the dataset that exhibited similar responses (see Supplementary Figure S1, which shows three instances of this relationship).

Furthermore, we have observed a strong linear correlation between the radiation patterns of different frequencies (on the same cut), indicating that the radiation pattern varies smoothly as the frequency changes (see Supplementary Figure S2). We utilized this observation while designing our surrogate solver to determine the radiation pattern at other frequencies within the range of our interest through linear interpolation. More detailed description of this observation can be found in Supplementary Section S1.4.

### 2.2 Basic antenna design

Infusion of the designer’s knowledge into the inverse design process not only ensures that the design process is grounded in practicality and real-world applicability but also significantly reduces the size of the required dataset. This is important particularly due to the complex nature of plasmonic systems, where generating large datasets is time-consuming and requires computationally expensive numerical simulations. Moreover, by integrating domain-specific knowledge, the network becomes more efficient at generalizing from smaller datasets. Here, to inject the designer’s knowledge into the model, the basic structure of the antenna is designed using the well-known formulas for plasmonic patch antennas [26], [27], and two separate datasets with three and four parameters have been generated by adding T-stub configurations to the predesigned basic structure.

In the optical regime, metals behave differently than in the radio frequency (RF) due to the negative values of the real part of their permittivity. This unique feature of metals enables them to support surface modes at the metal/insulator interface, namely the surface plasmon polaritons (SPPs). A plasmonic MIM waveguide is comprised of two metal/insulator interfaces where each metal/insulator interface supports individual SPPs. Bringing the two interfaces to the same proximity results in the coupling of the SPPs on the two interfaces and a single propagating plasmonic mode in the MIM waveguide. The propagating mode in a MIM plasmonic waveguide is a transverse magnetic (TM) in nature, and as a result one must either use modified propagation constants and impedances to model/analyze the problem using the conventional transmission line theory [67], or use mere approximative methods (see Supplementary Section S2) by taking advantage of the fact that the magnitude of the TM component is rather small with respect to the transverse component and approximate the mode as a TEM to design the basic parameters (it should be noted that the formulas and the methodologies outlined in Supplementary Section S2 are predominantly based on empirical and numerical fits that were previously proposed in the literature for plasmonic patch nanoantennas, and these methodologies are applied here exclusively for the design of the basic structure of the antenna which we will be used to generate the dataset, while our proposed inverse design framework will do further designs).

**Figure 2:**
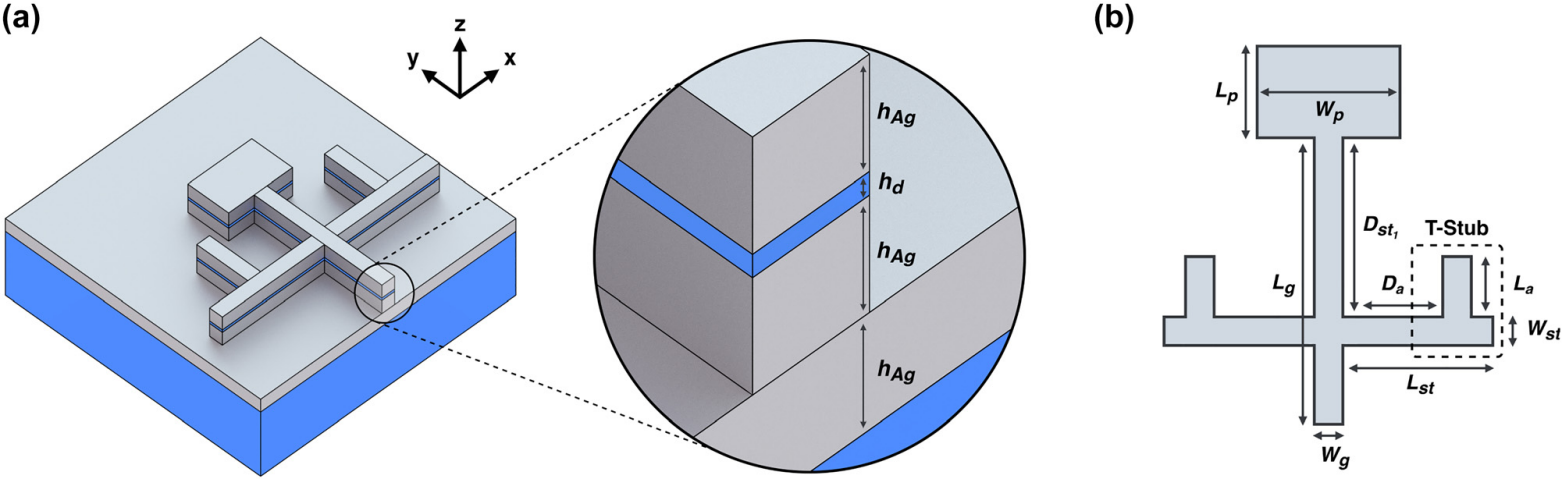
Basic structure of the plasmonic patch. (a) 3D view of the full structure, (b) top view of the antenna.

The basic parameters of the patch are chosen as shown in Figure 2, where *W*_*g*_ = 100 nm, *L*_*g*_ = 1,000 nm, *L*_*st*_ = 850 nm, *W*_*p*_ = 500 nm, and *L*_*p*_ = 320 nm. Our inverse network will determine *D*_*st*_, *D*_*a*_, and *L*_*a*_. This selection aims to achieve impedance matching for single band, dual band, and broadband operations, using various shapes and configurations of the T-stub that will be further designed and added to the structure by our inverse design network. The thickness of the metallic layers in the waveguide and the patch have been chosen in a way to be larger than the surface wave skin depth *δ*_*m*_ (see Supplementary Section S3), but not very small which leads to fabrication implications (*h*_*Ag*_ = 100 nm), whereas the thickness of the dielectric layer is chosen as *h*_*d*_ = 20 nm (see Supplementary Section S4, Figure S3(b)).

Although the most prominent and most accessible method to control the resonant frequency of the patch is by controlling the length of the patch, a different approach has been chosen here. Here, the length of the patch is fixed, and we will tune the resonant frequency (resonant frequencies for dual band and broadband operations) of the patch using various symmetrical T-stub configurations. The basic shapes of the T-stubs are based on the T-shaped resonators that previously used in diplexers [68] and dual band transformers [69]. Here, we will show that, our network is capable of generating all of the desired responses (forward problem) and the devices (inverse problem) for all of the queries using two T-stubs (the three-parameter case), and a combination of two T-stubs with two normal stubs (stubs without additional arms) configurations (the four-parameter case), without changing the patch dimensions.

There are several reasons behind this choice: by altering the T-stub dimensions and locations, one can also control the antenna’s bandwidth without significantly altering other antenna characteristics, whereas changing the patch size will not provide the same level of control over bandwidth. Additionally, although one of the widely accepted methods to induce dual band or broadband operation in the patches is introducing slots in the patch, this will increase the radiating edge of the patch and lead to higher edge currents, resulting in increased spurious radiation and decreased radiation efficiency. Moreover, slots might lead to higher cross-polarization levels; they require tighter tolerances during fabrication (especially in plasmonic structures where feature sizes are extremely small) as precise slot dimensions and positions are crucial for achieving the desired multi band performance, and slots are also typically hard to design as they can introduce additional resonances, which may lead to unwanted harmonic radiations. Apart from the drawbacks mentioned above, inducing multi band or broadband operation in the antenna typically using slots requires the creation of slots of different shapes and sizes in the patch and this method hugely increases the number of parameters in the hyperparameter space (e.g., length, width, shapes, and location of the slots), and the data required to train the network.

### 2.3 Multi-head convolutional surrogate solver

As previously mentioned, the simulation of electromagnetic devices requires considerable computational resources and time. For instance, simulating a single nanoantenna can take over 20 s (with symmetric boundary conditions), even on a high-end computer. To facilitate this process, we have utilized deep neural networks to approximate the simulation function. Using deep neural networks as a surrogate solver exhibits several advantages: it significantly reduces computation time, performs tasks orders of magnitude faster, and enables parallel evaluation of multiple samples. Moreover, we can use backpropagation to compute the derivative of the simulation function, which is beneficial for optimization tasks.

The architecture of deep neural networks plays a vital role in their performance. Factors such as the number and type of layers and the activation function significantly impact the network’s ability to generalize to unseen data. Additionally, selecting an appropriate inductive bias, such as convolution over fully connected, can reduce the required training samples. We have conducted an extensive hyperparameter optimization (HPO) process to select the optimal network architecture and training parameters (e.g., learning rate, weight decay, batch size). This process involves sampling different configurations based on the predefined range of hyperparameters and a set of network configurations. Subsequently, the network is trained with the sampled configuration, its performance is evaluated on the validation set, and the best configuration is selected from the sampled configurations (each iteration of this process is called a trial). HPO can speed up the entire process with trial pruning and early stopping techniques, in which trials with less promising results are terminated earlier. The parameters considered in the HPO for sampling include the learning rate, regularization weight, batch size, configuration of fully connected layers (i.e., number of layers and neurons), whether to use convolutional layers, and their corresponding configurations.

To train the network during each trial, mean-squared error is used to measure the error between predicted and actual responses. To reduce the computational cost of this step, only the *S*_11_ response is utilized during the hyperparameter optimization process, and the radiation pattern is employed only after the HPO trials. We use the Adam optimizer to learn the network weights, and the maximum number of epochs for the training of each trial is 1,000.

**Figure 3:**
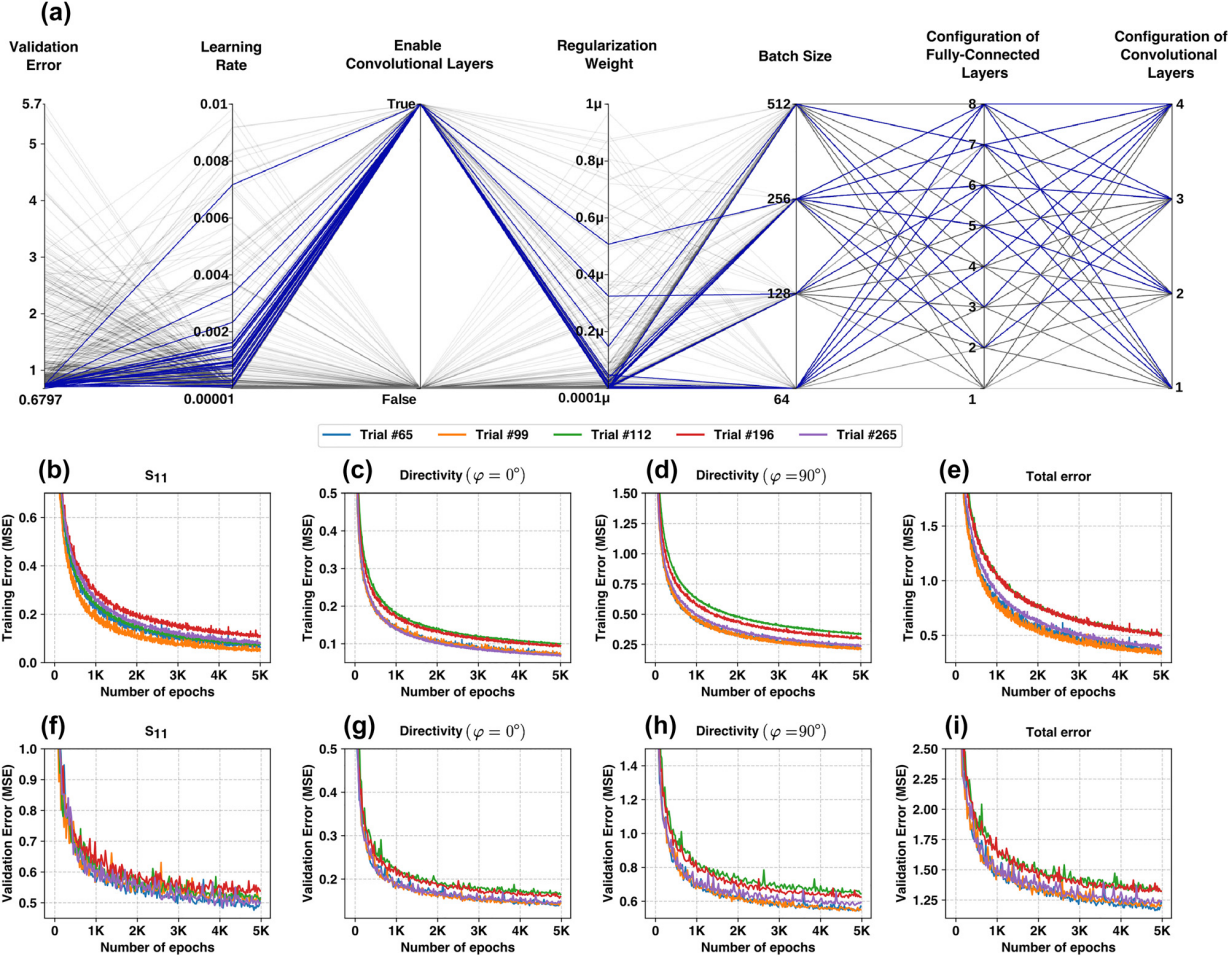
Hyperparameter optimization of the proposed convolutional surrogate solver. a) A parallel coordinate diagram shows the generated trials and their configuration during the hyperparameter optimization (HPO) process. The blue lines indicate the top 20 trials with the lowest validation error. (b–i) The training and validation learning curves of the top five trials generated by HPO. (b) Training error of *S*_11_, (c) training error of directivity (*φ* = 0°), (d) training error of directivity (*φ* = 90°), (e) total training error, (f) validation error of *S*_11_, (g) validation error of directivity (*φ* = 0°), (h) validation error of directivity (*φ* = 90°), and (i) total validation error.

Figure 3(a) illustrates a parallel coordinate plot of the generated trials (300 trials have been generated in the HPO process), showing the sampled configurations and their corresponding validation errors. The diagram highlights the 20 trials with the lowest validation error (*S*_11_), where all trials have a learning rate between 0.002 and 1*e* ^−5^, use convolutional layers, and their regularization weight is less than 2*e* ^−7^. We have selected the top five trials among the generated trials and trained them on the gathered dataset for longer epochs with the rest of responses. Figure 3(b)–(i) display the training and validation learning curves for this process. The curve indicates the successful convergence of models after 5,000 epochs without showing overfitting. We have selected the trial with the lowest validation error and evaluated the model on the test set to determine the model’s overall accuracy and to ensure that the model does not overfit the validation set.

The selected architecture, depicted in Figure 1(d), comprises two main components: the backbone and the convolutional blocks. The backbone block takes the parameters of the device as the input and maps them into the latent space. This block comprises five fully connected layers, each with 512 neurons. The last fully connected layer is followed by three convolutional heads that map the features from the latent space to the response space. The responses predicted by each convolutional head are *S*_11_ and radiation pattern in *φ* = 0° and *φ* = 90° planes, respectively. We have realized that a single head is enough to predict all the radiation patterns of different frequencies for each cut, due to the high linear correlation of the radiation patterns in the same cut. Since the radiation pattern changes gradually with frequency, a linear interpolation is utilized to approximate the radiation pattern at any frequency between 185 THz and 198.5 THz using the four approximated patterns from our surrogate solver. Given a device *d*, the trained surrogate solver is capable of estimating device responses $\hat{r}=\hat{f}\left(d\right)$ , where $\hat{r}$ comprises both the estimated *S*_11_ and radiation patterns in *φ* = 0° and *φ* = 90° planes, presented in concatenated vector form. Furthermore, the radiation patterns are interpolated at the specified frequency.

Due to the presence of spatial correlation in all responses, we designed the heads with convolutional layers instead of fully connected layers to effectively model spatial correlations and generate the final responses. Subsequently, during hyperparameter optimization, it was observed that convolutional layers consistently outperformed fully connected layers in capturing spatial correlations (see Supplementary Section S5 for additional information regarding the configuration of the convolution blocks).

We have evaluated the overall accuracy of our network using the test set. The following are the mean squared errors for each response: the total error for *S*_11_, the radiation pattern in *φ* = 0°, and *φ* = 90° planes is, 0.53, 0.16, and 0.6, respectively. It is worth mentioning that the current antenna structures have one plane of symmetry and the patterns are symmetric in *φ* = 0° while nonsymmetric in the *φ* = 90° plane, leading to a higher error of the radiation pattern in *φ* = 90° plane.

**Figure 4:**
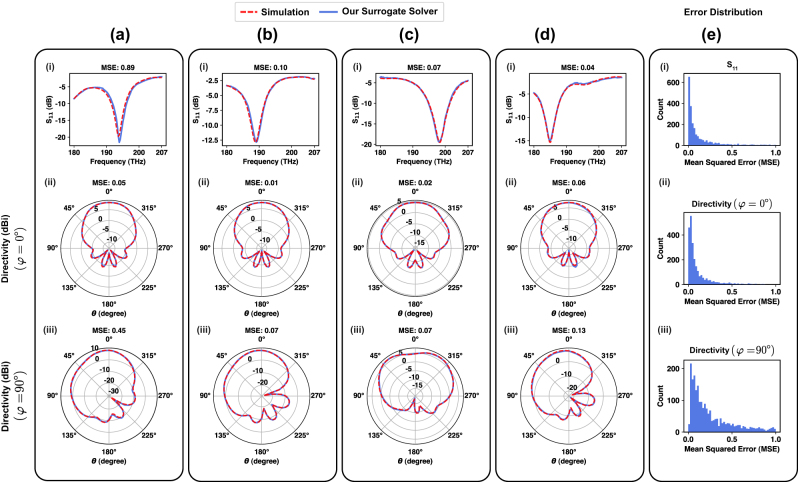
The prediction accuracy and the error distribution of the proposed surrogate solver. (a–d) The simulated response and the predicted response of four devices, (e) the error distribution of each response type.

Figure 4(a)–(d) depict the qualitative prediction accuracy of the network. These figures show the simulated and predicted responses generated by the surrogate solver for four devices, demonstrating an almost perfect match between the two. To better illustrate the distribution of errors in each response, we have computed the error distribution plot as shown in Figure 4(e), where results indicate that the majority of the test samples exhibit errors <1.0. More precisely, 90.84 % of the samples have *S*_11_ error <1.0, 97.64 % of the samples have directivity (*φ* = 0°) error <1.0, and 85.68 % of the samples have directivity (*φ* = 90°) error <1.0. The numerical value of the MSE in each sample may not accurately reflect the quality of alignment between the predicted and target responses. This is due to the fact that, responses can have very large dips (for example, *S*_11_ can have a dip value of −50 dB). A small difference between the predicted and target responses near the dip region may lead to a high squared error, despite the excellent visual alignment between the patterns, as shown in Figure 4. The MSE is primarily used for training purposes and to demonstrate the convergence of the process.

### 2.4 Inverse design framework

In our proposed framework, we have utilized the pseudo-inverse function to model the inverse problem. This approach offers several advantages, such as preserving one-to-many mappings, enabling the generation of multiple diverse designs (explained in 2.4.2), imposing fabrication constraints, and utilizing query-based objective functions (described in 2.4.3). The ultimate objective of our inverse design framework is to identify a set of candidate devices *D* that satisfy the pseudo-inverse condition, given a desired response *r*:

(3) $$D=\left\{d\in R^{k},{\left\|\hat{f}\left(d\right)-r\right\|}^{2}<\varepsilon \right\},$$

To achieve this, we have utilized the Particle Swarm Optimization (PSO) algorithm in combination with our neural network-based surrogate solver to efficiently explore the reduced device space ( $R^{k}$ ) and generate possible solutions for *D*. PSO is a population-based, meta-heuristic, evolutionary algorithm that is widely utilized in search and optimization problems and has proven as a highly effective approach for finding optimal solutions that minimize the objective function. The significant superiority of PSO over other alternatives such as genetic algorithm [70], apart from its straightforward implementation and accelerated convergence, lies in the memory retention of particles and the dynamic information exchange between them (information flow) [71], [72].

PSO starts by randomly creating particles to form a population in which each particle represents a unique configuration of a nanoantenna. In the next step, the population is evaluated using a predefined objective function (this evaluation is carried out simultaneously for all the particles using the surrogate solver). Consequently, particles are moved toward better solutions (with a lower objective function value) based on different factors, including the best local and global positions. The second and third steps are repeated iteratively until the particles are converged, and the results are used to determine a single optimum nanoantenna (Section 2.4.1) as well as multiple diverse nanoantennas (Section 2.4.2).

#### 2.4.1 Single optimal result

**Figure 5:**
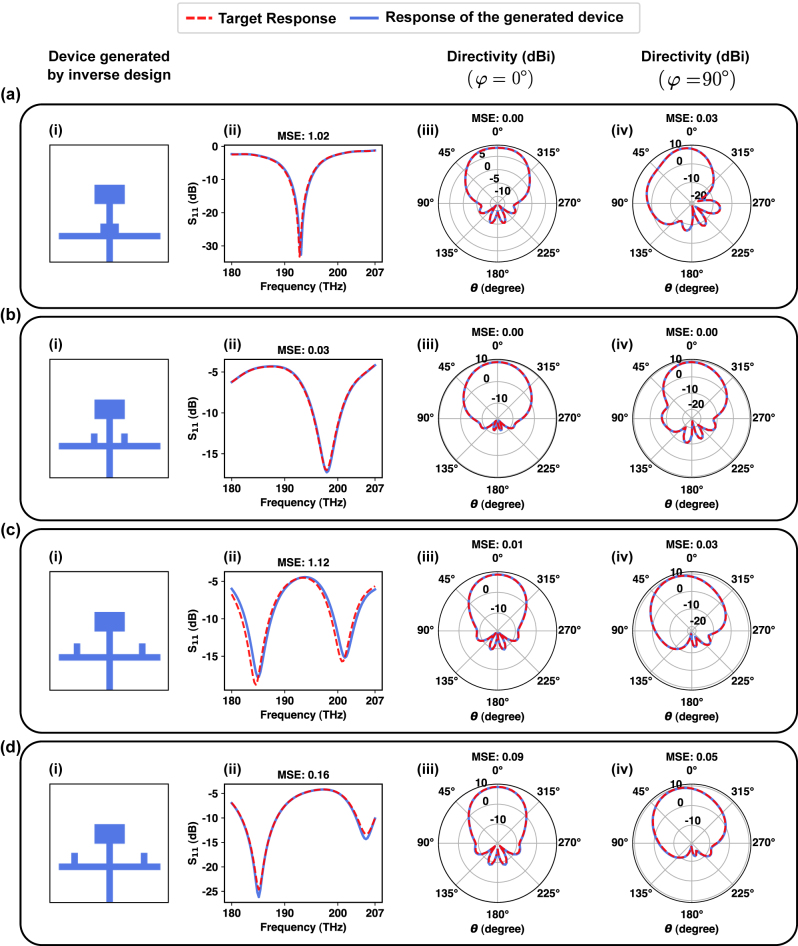
Inverse design verification experiment with the goal of generating single optimal devices given the target responses. (a–d) The generated devices by the inverse design framework given the target responses.

To obtain a single optimal device that meets the desired response, the particle with the lowest objective function value is selected after PSO has converged. To evaluate the performance of our inverse method in generating single results, we have tasked our network with generating a single configuration of a nanoantenna for 2,500 randomly sampled responses. The target *S*_11_ is defined across all frequencies (180 THz–198.5 THz), and the target radiation pattern is defined in *φ* = 0° and *φ* = 90° planes at four different frequencies. We have utilized the squared L2 distance as our objective function (discussed in 2.4.3), in which the entire shape of the generated and target responses must match. The target responses are extracted from the test set, consisting of randomly sampled devices with corresponding responses. In this experiment, we are confident that a device with the desired response exists within our design space. Our goal is to validate the capability of our inverse-design framework in finding these devices, also known as the physical targets [50], [51]. To quantitatively evaluate the performance of this experiment, we have calculated the mean squared error between the target and simulated responses of the generated devices. The total error for *S*_11_, the radiation pattern in *φ* = 0° and *φ* = 90°, is 0.46, 0.11, and 0.4, respectively. Figure 5 shows several instances of this evaluation where responses of the generated devices match very well with the target response (see Supplementary Section S6, Figure S7 for the error distribution for this experiment).

#### 2.4.2 Multiple diverse results

Due to the existence of one-to-many mappings in our dataset, a response may be realized with more than one device, creating several local minima in the optimization space. As PSO explores the design space, particles tend to get absorbed by regions where local minima are present. As a result, several clusters are formed after the convergence where the density of the particles is higher around local minima. In addition, using a query-based objective function brings flat regions into the optimization space, where several points meet the optimization criteria. To discover multiple diverse devices, the mean shift algorithm [73] is used to identify the clusters formed by PSO and locate the local minima. Mean shift is a nonparametric, density-based clustering algorithm used for segmentation and clustering, which can identify dense regions in the data space and finding local optima.

**Figure 6:**
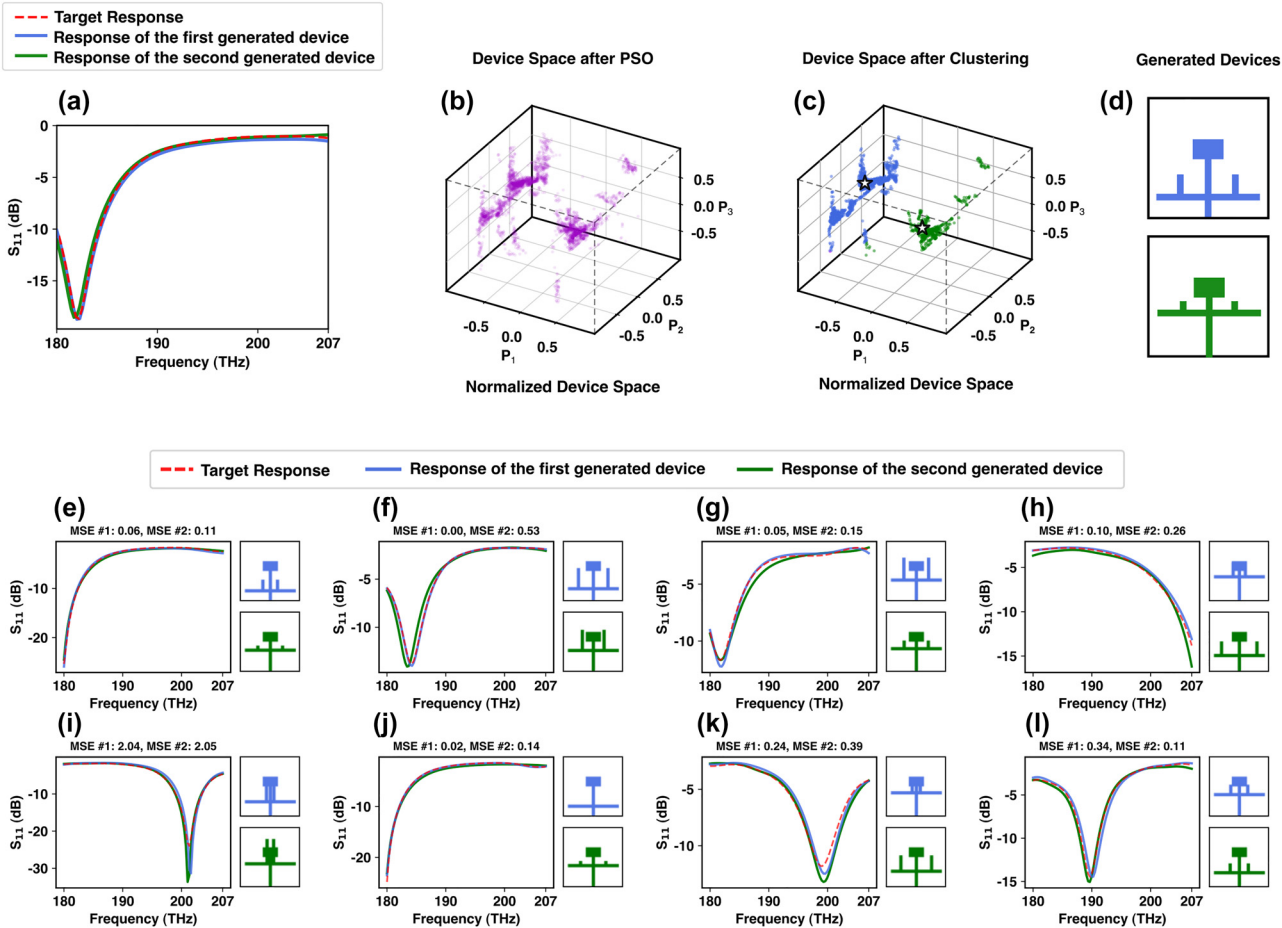
Qualitative results from the multiple diverse results experiment: (a–d) examples of utilizing a clustering algorithm (mean shift) to generate multiple diverse devices given a target response. (a) The response of the generated devices and the target one, (b) device space after being explored by the PSO with the purple dots showing the particles, (c) particles are clustered into two groups using the mean shift algorithm, (d) generated devices by inverse design. (e–i) Qualitative examples of the experiment where the proposed inverse design framework was tasked to generate multiple diverse devices given the target response.

Figure 6(a)–(d) illustrate the procedure of discovering multiple diverse designs using the PSO particles and the mean shift algorithm. To obtain a set of diverse results *D*, after exploring the device space using a neural network-based surrogate solver, the resulting particles are filtered based on the value of the objective function, the clusters are identified using the mean-shift algorithm, and a set of candidate devices is determined by selecting the best particle in each cluster.

An experiment has been conducted to evaluate the accuracy of our inverse method in generating multiple diverse results with the same goal as in the previous section, where the squared L2 distance is used, and the generated response’s shape must match the target response. However, in this evaluation, the inverse design framework is tasked with discovering more than one device for each target *S*_11_. The qualitative results of this experiment are shown in Figure 6(e)–(i), indicating that the inverse design framework successfully discovered multiple configurations given the target responses. We have further improved the performance of PSO in exploring multiple local minima by prioritizing exploration over exploitation. This was achieved by increasing the number of particles and selecting the ring topology over fully connected. In the ring topology, particles can only communicate with their nearest neighbors, which limit their perception of the global minimum. This encourages distributed exploration in which the optimization space is partitioned into multiple regions, and particles operate exclusively within those specific areas.

#### 2.4.3 PSO’s objective function

Two different objective functions have been utilized throughout our framework for the PSO, the squared L2 distance and the query-based objective function. The squared L2 distance between the predicted and target responses is defined as follows:

(4) $$l\left(d,r\right)={\left\|\hat{f}\left(d\right)-r\right\|}^{2},$$

where *r* encapsulates the entire *S*_11_ and the radiation pattern in the two radiation pattern cuts (at the specified frequency), all stacked together in a single vector. This type of objective function is useful when the generated response needs to precisely conform to the target response, i.e., to accurately match the target *S*_11_ in the entire working frequency and the radiation pattern at every direction in the specified frequency. This objective function is used to evaluate the performance of the inverse design framework, quantitatively and qualitatively (Sections 2.4.1 and 2.4.2). It is also convenient to perform the inverse design task merely by defining a few conditions on the target response that the generated device must satisfy. This simplifies the inverse task for the designer, as one does not need to provide the full definition of the target response but only a few desired conditions. As a result, the framework can generate a wider range of candidate devices.

To achieve this, we have introduced and employed a query-based objective function. The term query refers to a request sent to the inverse-design framework to generate a nanoantenna. It contains a set of high-level conditions that the generated antenna should fulfill. For instance, to generate an efficient single band nanoantenna, the generated device must satisfy the following conditions: having an *S*_11_ dip less than −10 dB at the antenna’s working frequency and directivity more than 10 dBi in a specific direction. Each condition in the query is modeled as a cost function that takes the predicted response and returns an error scalar based on how well the requirement is met. The query-based objective function is then defined as a weighted sum of the cost functions, which is used as the objective function for the PSO:

(5) $$l\left(d,r\right)=\underset{c\in C}{\sum }w_{c}c\left(\hat{f}\left(d\right),r\right),$$

where *c* is a cost function, *w*_*C*_ is the corresponding weight, and *C* is a set of cost functions of the specified query. We have used the query-based objective function to design a wide range of different nanoantennas, which can be found in the results section.

In our framework, we can handle fabrication constraints in the following ways: first, we can fix a set of device parameters in advance. For instance, we can task the framework to generate a device based on a desired response with a predefined stub location. Second, we can limit the range of motion of the parameters. For example, we can set a minimum and maximum value for the notch length. Finally, we can add an additional cost function to the PSO’s objective function to account for any fabrication limitations. This will output a cost if the constraints are not met.

## 3 Results

In this section, we will put our inverse design framework through a set of comprehensive tests to generate designs that not only satisfy the standard criteria required in real-world applications but potentially outstripping them from performance point of view, while addressing complex design challenges. In this set of query-based experiments, we are unsure if the desired device exists in the design space, and whether it is physically possible to have a device with such responses (also known as nonphysical targets [50], [51]). However, the inverse network generates the closest match that it can find in the design space.

### 3.1 Single band nanoantennas with maximum directivity

**Figure 7:**
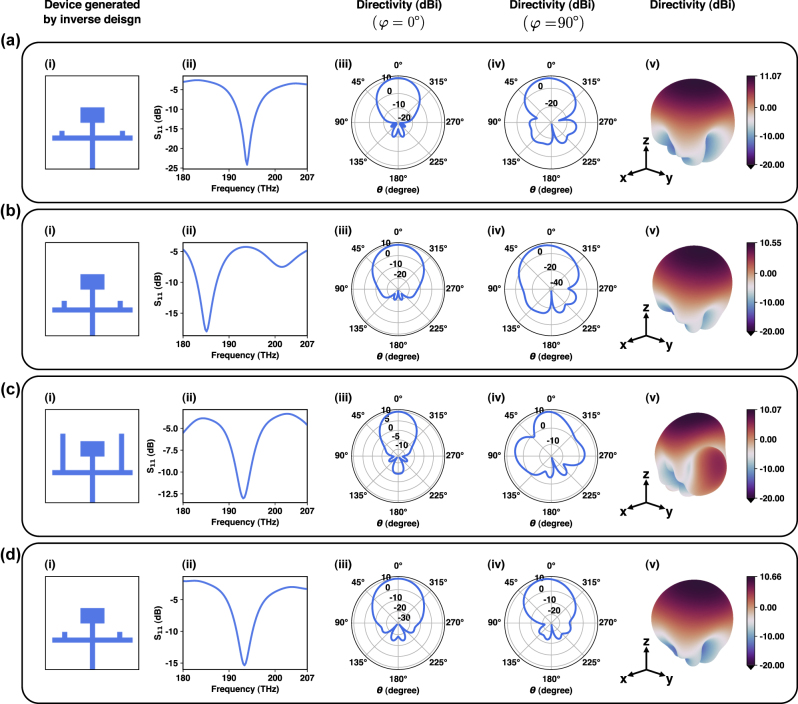
Single band nanoantennas designed by the proposed inverse design framework: (a) a single band nanoantenna (device *d*_1_) designed for *f* = 193.5 THz with *S*_11_ < −10 dB and the highest possible directivity in *φ* = 0° and *φ* = 90° planes. (b) A single band nanoantenna (device *d*_2_) designed for *f* = 185 THz with *S*_11_ < −10 dB, the highest possible directivity in *φ* = 0° and *φ* = 90° planes, and a suppressed radiation in *θ* = 180°. (c) and (d) Unconstrained and constrained single band nanoantennas (devices *d*_3_ and *d*_4_), respectively. Each subfigure (i–v) in panels (a–d) shows the schematic of the device, *S*_11_, directivity in *φ* = 0° plane, directivity in *φ* = 90° plane, and the 3D radiation pattern for each of the devices, respectively.

Typically, a single rectangular patch exhibits directivities in the range of 5–8 dBi; however, as shown in Figure 7(a), one can see that a directivity of up to 11.07 dBi is made possible for a single patch with our inverse design framework. In this section, the inverse design network has been tasked to design single band nanoantennas at frequencies *f* = 193.5 THz with *S*_11_ < −10 dB and the highest possible directivity in both *φ* = 0° and *φ* = 90° planes.

An interesting observation that can be made from the single band devices generated by our network (see Supplementary Section S6 for more single band devices generated by our framework) is that lengths of the arms of the T-stubs are mostly short in length. This makes perfect sense from a physical point of view because if the length of the arm of the T-stub were longer, it would be either closer to the patch or pass through it. Either of the cases distorts the fringing field at the beginning side of the patch, acting as if there were slots in the patch, creating multiple resonances and making the patch act as multiple cavities (multi band operation). This is because a patch acts as a resonant cavity and typically resonates when its physical dimensions correspond *λ*/2. Introducing slots into the patch modifies this resonant cavity by introducing discontinuities in the current distribution, changing the effective length of the antenna and distribution of the electric field across the patch, and the fringing fields, which is similar to creating multiple smaller resonators within the main patch (see Supplementary Section S6 for another set of the single band antennas).

It should be noted that the radiation efficiency of all of the single band nanoantennas generated by our inverse-design framework is plotted in Supplementary Figure S10(a), where radiation efficiencies of all of the nanoantennas lie approximately in the range of 70–75 % at their central operational frequency, illustrating high radiation efficiencies, given the plasmonic nature of the structures. Additionally, polarization of the radiated waves, for all of the single band antennas, are illustrated in Supplementary Figures S10(b)–(e) where the axial ratio, which is the ratio of the major axis to the minor axis of the polarization ellipse, is plotted against all *φ* and *θ* angles in 2D equirectangular maps, showing linear polarization around the *z*-axis perpendicular to the antenna plane where the radiation is maximum.

In integrated circuits, planar antennas with half-space limited radiation patterns are of great interest, as they inherently prevent interference with the electronic and photonic components underneath them. Here, we will aim for the back lobe suppression, and the inverse design network has been tasked to design nanoantennas at frequencies *f* = 185 THz with the highest possible directivity at *θ* = 0°, and minimum radiation at *θ* = 180°, in both *φ* = 0° and *φ* = 90° planes. The generated device and its responses are shown in Figure 7(b) (see Supplementary Section S6 for more instances of back lobe-suppressed single band nanoantennas).

### 3.2 Single band nanoantennas with constraints

One of the major strengths of the proposed framework compared to its counterparts is that, apart from the fabrication/design constraints that were already applied in the dataset generation phase, further constraints can be applied to parameters after the training process. This is of great importance in cases where the training process is finished; however, because of various design-specific, space-constraints, one wants to further limit the parameters. Typically, this process requires retraining the network again while considering these constraints; however, in our framework, this can be done without retraining the network and simply by adding a set of constraints during the inverse process (explained in Section 2.4.3).

As the first constraint, we will fix the length of the arm of the T-stub (as can be seen from a direct comparison between Figure 7(c) and (d)). This case is of particular importance in 2D arrays where the long length of the arm in the T-stub makes it difficult to have a dense array in the *y*-direction. To illustrate this point, we have first tasked the network to design an antenna with *S*_11_ < −10 dB and directivity >9 dBi at *f* = 193.5 THz. Let us consider the cases where our network has generated devices with long T-stub arms as shown in Figure 7(c). As mentioned before, if there exist multiple devices that generate the same results (multiple clusters), our algorithm has the capability to choose either of the clusters (the network can be configured to either choose the best response, or any of the other clusters depending on the defined criteria). As a result, we will ask the network to only generate devices, satisfying the exact same queries at the same frequencies (*S*_11_ < −10 dB and directivity >9 dBi at *f* = 193.5 THz); however this time, length of the arm of the T-stub is limited. The generated devices and their corresponding responses for the unconstrained and constrained cases are shown in Figure 7(c) and (d), respectively, perfectly illustrating the capabilities of our inverse network and the fact that, in order to impose constraints on the design, there is no need to retrain the network, thereby constraints can be applied even after the training process is finished (see Supplementary Section S6 for the second case of imposing constraints where we will fix the location of the arm of the T-stub at 50 nm and the network is tasked to generate devices with *S*_11_ < −10 dB and directivity >8 dBi at *f* = 186.5, 187.5, 189.5, 193.5, and 196.5 THz).

### 3.3 Dual band and broadband nanoantennas

**Figure 8:**
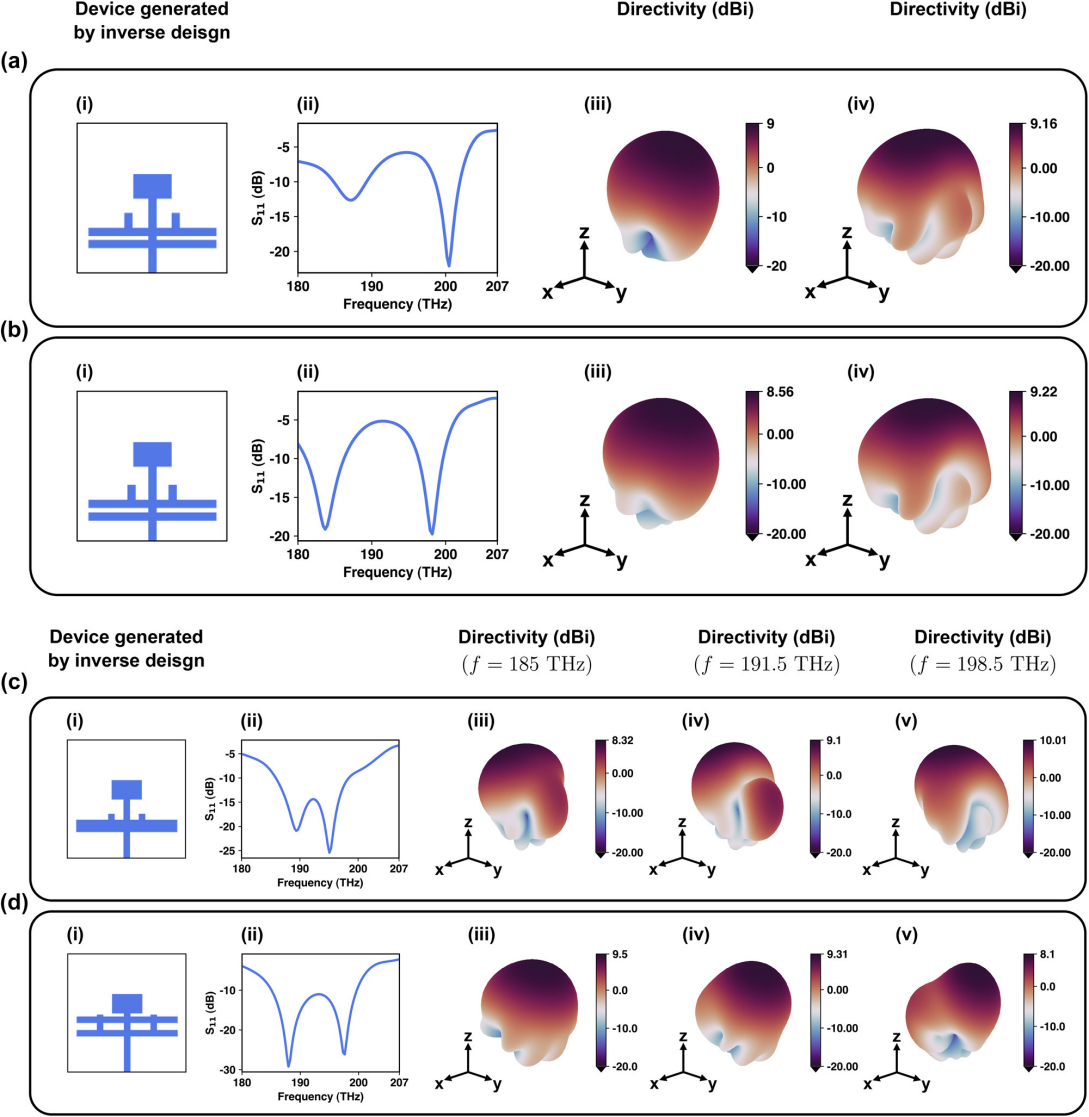
Dual band and broadband nanoantennas designed by the proposed inverse design framework: (a) a dual band nanoantenna designed for *f*_1_ = 188 THz and *f*_2_ = 201 THz and (b) a dual band nanoantenna designed for *f*_1_ = 185 THz and *f*_2_ = 198.5 THz with *S*_11_ < −10 dB and the highest possible directivity in *φ* = 0° and *φ* = 90° planes. (c) and (d) Two broadband nanoantennas with bandwidths of 13.5 THz and more than 22 THz, respectively. Subfigures (i–iv) in panels (a) and (b) show the schematic of the device, *S*_11_, and directivities in the frequency dips of each device, respectively. Subfigures (i–v) in panels (c) and (d) show the schematic of the device, *S*_11_, and directivities in *f* = 185 THz, *f* = 191.5 THz, and *f* = 198.5 THz, respectively.

In this test, the network is tasked to generate dual band nanoantennas operating at two uncorrelated frequencies with *S*_11_ < −10 dB and directivity >8 dBi at both frequencies. We have tasked the network (as shown in Figure 8(a) and (b)) to specifically generate dual band antennas with two uncorrelated frequencies because uncorrelated frequencies do not share harmonics or other signal characteristics that can lead to cross-band interference, thereby they are less likely to interfere with each other. Moreover, since the two operational frequencies do not interfere with each other, they can be used simultaneously without degrading each other’s performance, which leads to better spectrum efficiency. From the optical imaging point of view, dual band antennas can capture images at two different wavelengths simultaneously, providing richer information about the subject which is of huge interest in microscopy. Additionally, since different wavelengths interact differently with various objects and materials, utilization of dual band antennas in LiDARs enables them to operate at two different wavelengths, leading to an improve in the resolution, accuracy, and better differentiation between different types of objects or materials.

Bandwidth plays a key role in the capacity of optical intra-/interchip communication networks as broadband nanoantennas are capable of transmitting/receiving signals through multiple channels. As mentioned before, patch nanoantennas are inherently resonant structures and their impedance changes rapidly with frequency, leading to a large mismatch between the patch and the feed, resulting in their narrowband operation. As a result, having broadband plasmonic patch nanoantennas is of great importance in photonic integrated circuits due to their low-profile, planar nature. As the last test, we have tasked our network, as shown in Figure 8(c) and (d), to design broadband nanoantennas with *S*_11_ < −10 dB and directivities >8 dBi over the whole range. This superior broadband feature while maintaining relatively high directivities over the whole range enables the plasmonic patch nanoantennas to play a pivotal role in photonic integrated circuits.

### 3.4 Multiple-diverse results

**Figure 9:**
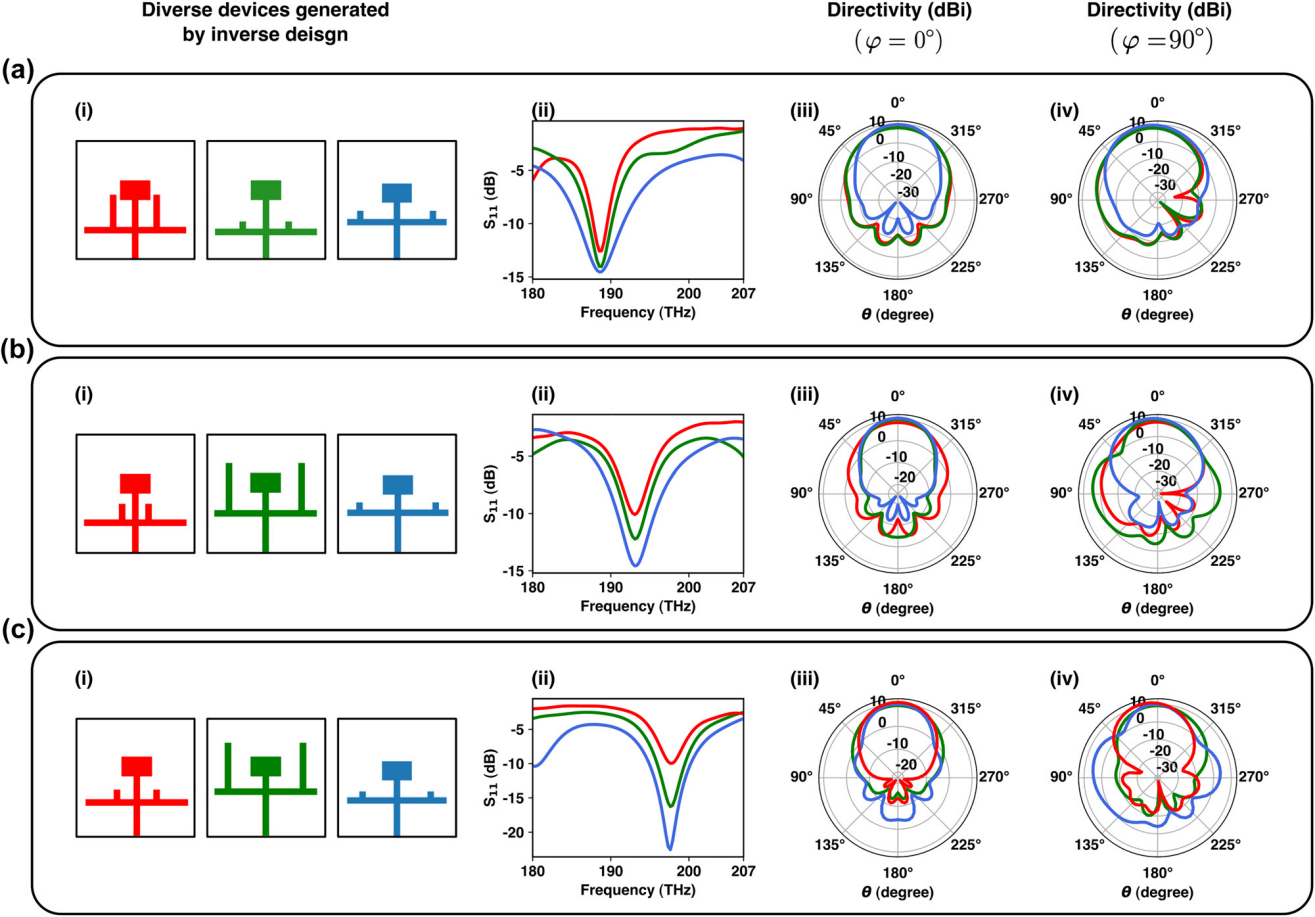
Multiple diverse single band nanoantennas designed by the proposed inverse design framework: (a)–(c) multiple diverse devices generated for the same query. Subfigures (i–iv) in (a)–(d) represent (i) three different nanoantennas generated for the same query, (ii) their corresponding *S*_11_, (iii) directivity in phi, and (iv) directivity in phi = 90 planes, respectively. Red, green, and blue plots in each subpanel (ii–iv) correspond to the nanoantenna of the same color.

In this section, our inverse design framework is tasked to generate three different devices for each set of defined criteria, shown in Figure 9(a)–(c), respectively. For instance, Figure 9(a) illustrates three different devices that have been generated by our inverse-design framework with *S*_11_ < −10 dB, and directivity >10 dBi in both *φ* = 0° and *φ* = 90° planes, at *f* = 187.5 THz. Figure 9(b) and (c) also show three different devices generated by our inverse network, with the same criteria, but this time at *f* = 193.5 THz and *f* = 198.5 THz. As it is obvious in Figure 9, the proposed framework is capable of successfully generating multiple devices for a set of criteria, each of which can be used for different applications and according to different design limitations.

## 4 Discussion

In this section, we will discuss the nonlinearity of the current problem, and how the proposed method can be extended for use in inverse design of random media where extremely large datasets are required.

### 4.1 Discussion on nonlinearity of the problem

Although the parameter space (3 and 4 parameters) may seem small at the first sight, the amount of nonlinearity they introduce to the result space is significant. In our work, the combination of stubs and their arms serve as the impedance matching network, either for single band, dual band, or broadband cases. The nonlinearity of the problem is partially embedded in the context of impedance matching and the fact that the input impedance of each section with a length *l* shows a tan(*βl*) or cot(*βl*) dependency, which itself is the source of nonlinearity of the problem because of the nonlinear behavior of the tangent function near its poles (in various certain regions a slight change in a parameter leads to a huge change in the response). On the other hand, addition of the arm on a stub introduces another level of frequency sensitivity: at the junction of the arm and the stub, the input impedance depends on both tan(*βl*_1_) and tan(*βl*_2_), and as a result, location of the arm and its length introduces a relatively significant amount of nonlinearity in certain regions of the input impedance function at the junction of the stub and its arm (see Supplementary Figures S13), showing two examples of this nonlinearity, where a rather slight change in the location of the arm of the T-stub (*D*_*a*_) leads to emergence of nonlinear changes in the *S*_11_. Furthermore, adding the second stub (as in the 4-parameter case) results in a new impedance transformation path on the Smith chart, which increases the nonlinearity (since with combination of two stubs, the impedance point can traverse a potentially looping or crisscrossing through different reactance and resistance levels paths). All of the mentioned nonlinearities combine with another significant source of nonlinearity of the problem, which is the coupling between different sections of the structure such as the coupling between stubs, T-stub’s arm, and the patch.

### 4.2 Novelty of the current framework and its potential for extension to problem consisting random media

It is important to briefly mention the novelties of the framework and why the proposed approach is a good candidate for use in random media, such as deep tissue imaging [74], random lasers [75], study of coherent backscattering [76], [77], quantum information processing [78], and random metasurfaces [79].

Nonuniqueness nature of the inverse problem, where a response can be realized by multiple devices, makes it challenging to directly learn the inverse mapping using discriminative neural networks. Approaches, such as tandem networks [58], aim to reduce the one-to-many mappings to a one-to-one mapping in order to learn the inverse mapping directly. However, this may result in eliminating devices from the device space that could still be useful. It is important to generate multiple devices because it allows the designer to choose a device that exhibits less sensitivity to fabrication imperfections. When two devices have the same desired response, the one that is more stable and less sensitive to fabrication is preferred. Generative approaches, including those based on variational autoencoders [60], [61] and generative adversarial networks [43], [62]–[64], have the ability to generate multiple devices. However, due to the complexity of training, these approaches may still suffer from mode collapses and fail to adequately capture the diversity of the device space.

An important capability of our network that renders very useful for inverse design of random media is preserving the one-to-many mappings, which is crucial for capturing the inherent complexity of the problem as it enriches the dataset for training of the network, leads to better generalization and prediction capabilities, and facilitates an effective and comprehensive exploration of the design space, leading to discovery of optimal solutions that might otherwise be overlooked. Additionally, many of the output devices may not be exactly realizable due to various factors (such as environmental variations in real-life scenarios, special arrangements of scatterers that might be hard to fabricate, etc.), as a result preserving the one-to-many mappings will handle this issue effectively by offering multiple alternative solutions. Moreover, in real-life random media scenarios, measurement noise and statistical fluctuations in the arrangement of scatterers can lead to discrepancies in the design process and performance. Preserving the one-to-many mappings mitigates the impact of such noises/fluctuations by offering alternative solutions.

Additionally, the proposed approach allows for adjustments and optimizations by applying post-training constraints, which is of great importance, especially in random media where many of the output devices may not be fabricable and the applications of further constraints are mandatory (which can be done without any retraining procedure using our network). This means that if a device with a desired response exhibits different, unwanted behavior after fabrication due to errors in the fabrication process (for example, the feed and arm of the T-stub being merged due to fabrication inaccuracy), we can ask the framework to find another device with the same response but less sensitive to the fabrication imperfections. For instance, this can be achieved by fixing a parameter in the PSO search space, limiting the range of motion of the parameters, or by defining an additional cost function in the PSO objective function. In tandem networks [58], one-to-many mappings are eliminated, meaning that for each response, only one device can fulfill it, making it impossible to have other devices that meet the fabrication constraints. In generative networks also, this aspect has not been explored, especially in approaches with larger degrees of freedom that generate freeform objects, leading to devices that are not fabricable.

The proposed architecture has been developed to learn from smaller dataset sizes and presents a relatively good level of generalization (considering the nonlinearity of the problem mentioned earlier in Section 4.1) and is a good candidate for utilization in inverse design problems concerning random media with a large number of parameters, where extremely large datasets are required for the network to learn the relationships in those highly nonlinear spaces.

It is important to note that in generative approaches and tandem networks, the inverse function *f* ^−1^ : *r* → *d* is directly modeled, where the entire response $r\in R^{672}$ is required to be provided to generate a device. However, this may not be favorable as only the response in a specific region $r\in R^{q}$ might be of interest (*q* ≪ 672), and providing arbitrary responses in the rest of the regions may limit the diversity of the generated devices to those that exactly exhibit $r\in R^{672}$ . In our framework, we can use a query-based approach to search for the desired device simply by defining a few conditions instead of providing the entire response. This results in finding additional devices that exhibit the desired behavior.

### 4.3 Importance of using PSO as the search algorithm

PSO and GA [70] are the most prominent optimization algorithms used extensively in numerous applications due to their versatility, robustness, and their ability to navigate through complex, high dimensional problems. Apart from PSO’s simplicity, fewer tuning parameters, and faster convergence speed in rugged and complex spaces, in the following, we will discuss multiple reasonings behind the choice of PSO over GA for our network.

The most significant advantage of PSO over other evolutionary algorithms, such as GA, lies in the inherent memory of the particles and the information flow between them. Each particle in PSO has a memory and shares its own experience with all other particles while obeying universal rules. Consequently, each particle benefits from other particles’ knowledge, enabling the swarm to efficiently navigate through complex and rugged spaces collectively. Moreover, PSO does not rely on gradient information, making it a good candidate for problems with highly nonlinear, nondifferentiable, or noisy objective functions.

In PSO, each particle’s position is updated based on its personal experience and its knowledge of the global experience shared by other particles, which will result in a balance between exploration and exploitation by preventing premature convergence in highly nonlinear spaces. This is in a strong contrast with GA, which relies on random discrete crossover and mutation operators and struggles to maintain this balance, leading to overexploitation or insufficient exploration of the search space. This is due to the fact that crossover operators may produce offsprings without inheriting the useful features of parents, leading to poor exploration, whereas mutation often comes at the cost of disrupting the existing good solutions, making it very challenging to find the optimum(s) in problems with rugged spaces. The discreteness of the crossover and mutation operators in GA also introduces abrupt changes in the search space and may lead to fluctuations in the errors, contrary to PSO, which benefits from a smoother search trajectory due to the continuous nature of adjustment of particle velocities.

Furthermore, over successive generations, GAs tend to lose population diversity, especially in cases where selection pressure is high. This loss of diversity mitigates the algorithm’s ability to escape local minima, which is more pronounced in highly nonlinear problems.

## 5 Conclusions

In this study, we have developed an efficient framework for the inverse design of plasmonic patch nanoantennas in the NIR regime. Our framework can design a wide range of devices including single band, dual band, and broadband antennas, with directivities of up to 11.07 dBi and radiation efficiencies reaching 75 % for a single patch. Moreover, our approach demonstrates a remarkable versatility in terms of applying various post-training design and application-specific constraints where, in addition to the primary fabrication constrains that have been considered while generating the dataset, further constraints can also be applied after the training process. This is crucial in addressing the ever-expanding needs of modern optical phased arrays, where designers are dealing with increasingly strictly stringent integration requirements. The proposed approach preserves the one-to-many mappings and provides the designer with the ability to choose from multiple diverse designs, given specific geometry and constraints. Our approach takes a significant departure from traditional NN-based inverse-design methods and sets a precedent for future research in the field leveraging the robust predictive and generative capabilities of deep neural networks in optical designs. This paradigm shift toward an inverse design approach fosters a more efficient and creative design process, enabling the exploration of innovative optical designs that might be overlooked or infeasible in conventional forward and inverse-design methods.

## Supplementary Material

Supplementary Material Details
