# Supplementary material for: Integrating deep convolutional surrogate solvers and particle swarm optimization for efficient inverse design of plasmonic patch nanoantennas
Source: Nanophotonics. 2024 Aug 2;13(21):3963–83. doi: 10.1515/nanoph-2024-0195 (PMC11501072; doi:10.1515/nanoph-2024-0195)
Supplement: Supplementary file 1 — Supplementary Material Details [file j_nanoph-2024-0195_suppl_001.pdf]

Supplementary Material

Integrating Deep Convolutional Surrogate Solvers and Particle Swarm Optimization for Efficient Inverse Design of Plasmonic Patch Nanoantennas

Saeed Hemayat,[a†] Sina Moayed Baharlou,[a,b†] Alexander Sergienko,[b] and Abdoulaye Ndao[a,b*]

[*] Corresponding author: Abdoulaye Ndao, Department of Electrical and Computer Engineering, University of California, San Diego, La Jolla, CA 92093, USA; e-mail: a1ndao@ucsd.edu

[a] Department of Electrical and Computer Engineering, University of California, San Diego, La Jolla, CA 92093, USA

[b] Department of Electrical and Computer Engineering and Photonics Center, Boston University, 8 Saint Mary's Street, Boston, MA 02215, USA

[†] Equal contribution

The supplementary file is organized as follows:

S1 Dataset Generation and Analysis
S2 Basic Antenna Design
S3 Alternative Approach to Model the MIM Structures
S4 Obtaining Widths of the Patches
S5 Proposed Architecture and Hyperparameter Optimization
S6 Extended Results
S7 Nonlinearity of the problem
S8 Comparison to lookup table algorithm

S1 Dataset Generation and Analysis

1.1 Latin Hypercube Sampling

Latin hypercube sampling (LHS) [1], [2], [3] behaves mostly similar to stratified sampling and efficiently reduces the number of runs required for reliable estimation of the behavior of a system. In each dimension, a value is randomly selected from each interval with a critical condition that once a value is selected from a specific interval, same interval cannot be chosen again (also known as sampling without replacement). The selected values from each dimension must be combined afterwards to form a unique sample point. Putting the above explanations in a mathematical formalism, for each dimension $i = 1, 2, ..., n$, the whole interval is divided into m equal subintervals

$[(j-1)/m\,,j/m]$ (where j = 1, 2, ..., m) with a length of 1/m. Assuming dimension i and subinterval j, a random point x_{ij} is selected according to the following equation:

$$x_{ij} = \frac{j+r_{ij}-1}{m}, \tag{S1}$$

where rij is a random number in the interval range. In order to create a single point across all dimensions, one must combine the stratified (equally probable) intervals with the random permutations to select one point from each interval. A point pk, as a unique combination across all dimensions can be generated considering ξ_i, a random permutation of {1, 2, ..., m} in the ith dimension using the following equation:

$$p_k = \left(x_{1\xi_{1(k)}}, x_{2\xi_{2(k)}}, \dots, x_{n\xi_{n(k)}}\right), \tag{S2}$$

where k = 1, 2, ..., m. It can be seen that, a random permutation of the m intervals for each of dimensions will be generated independently for each dimension which means that each dimension will have its own unique ordering of intervals.

LHS guarantees that each subinterval contributes just one sample per each dimension, ensuring that samples are spread evenly over the entire space and not clustered together as they might in simple random sampling. It should be noted that, LHS takes advantage of a far efficient approach as it does not require as many samples to fill the space, compared to the random sampling. This is due to the fact that LHS inherently ensures the coverage across all dimensions, contrary to random sampling in high-dimensional spaces, as the volume increases exponentially with addition of dimensions.

1.2 Dataset with four parameters

To generate the dataset with four parameters, we followed the same process as we did for the dataset with three parameters. This dataset is comprised of 100,000 samples where 90% of the data

has been used for the training (90,000 samples) and the remaining 10% has been used for validation and testing (5,000 samples each).

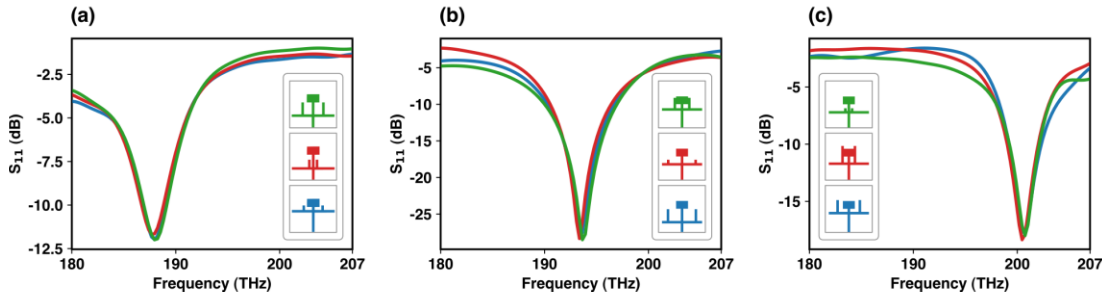

Figure S1. Illustration of the one-to-many mappings present in the dataset. (a-c) depicts three instances in which three distinct devices exhibit a similar S_{11} response.

1.3 One-to-many mappings

Figure S1 illustrates three samples showing the existence of one-to-many mappings in dataset with three parameters.

1.4 Linear Correlation

Pearson's Product-moment correlation is used here to determine the correlation between the radiation pattern cuts at different frequencies. The radiation pattern of the samples at each frequency can be considered as a multi-dimensional random variable. Therefore, to determine the overall correlation, we have calculated Pearson's coefficient for each dimension individually and took the average across all dimensions afterward. Figure S2 displays the determined correlation matrix, where each entry shows the averaged Pearson's coefficient between two cuts at different frequencies. The results indicate a strong correlation between the radiation pattern cuts in $\varphi = 0^{\circ}$ and those at $\varphi = 90^{\circ}$ planes.

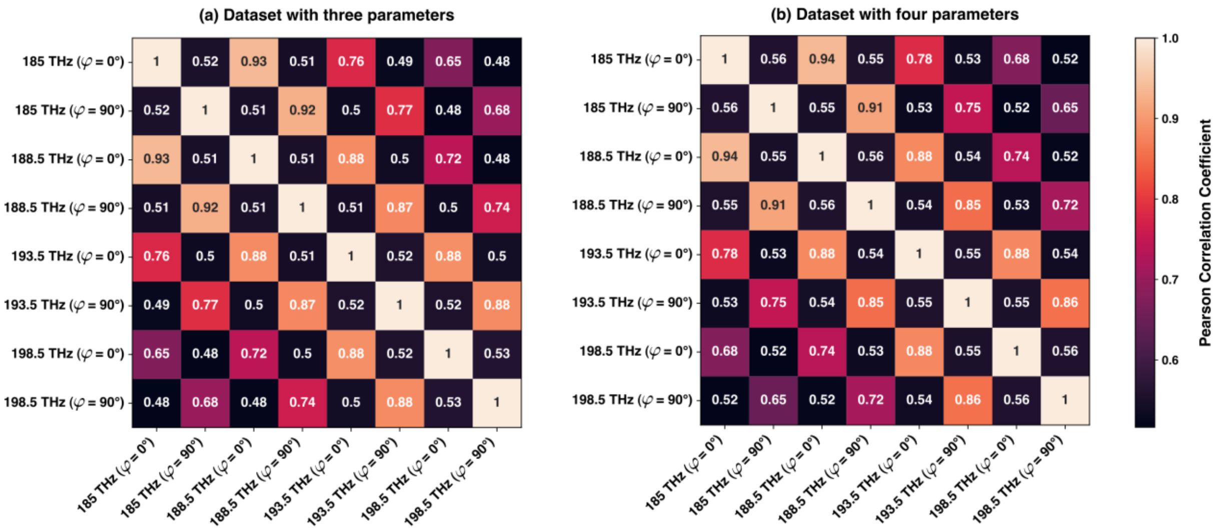

Figure S2. The correlation matrices showing the strong linear correlation between the radiation patterns at different frequencies in the same cut. (a) correlation matrix computed on the dataset with three parameters, (b) correlation matrix computed on the dataset with four parameters.

S2 Basic Antenna Design

The propagating mode in a an MIM plasmonic waveguide is a TM mode, and as a result (assuming the dielectric layer is spanned over $\frac{h_d}{2} < z < \frac{h_d}{2}$ region as shown in Figure 2 of main manuscript), one can write the magnetic field as the following [4]:

$$H_x = \begin{cases} Ae^{j\beta y+\alpha_1 z}, & z < -\frac{h_d}{2} \\ Be^{j\beta y+\alpha_2 z} + B'e^{j\beta y-\alpha_2 z}, & -\frac{h_d}{2} < z < \frac{h_d}{2}, \\ A'e^{j\beta y-\alpha_1 z}, & z > +\frac{h_d}{2} \end{cases} \quad \text{(S3)}$$

where, β is the propagation constant of the propagating plasmonic mode, $\alpha_i = \sqrt{\beta^2 - {k_0}^2\varepsilon_i}$ is the decay constant in each layer, and ε_i is the permittivity of the corresponding layers. The present analysis serves only to constitute an approximate relationship. Without loss of generality, we will assume the mode is symmetric which implies $A = A'$ and $B = B'$. The coefficient $B(B')$

can be determined using the boundary conditions, however we will not calculate it here since it is the shared factor in both magnetic and electric fields (as shown in the following) and will be eliminated in the calculation of the impedance. Considering the magnetic field in the insulator region and assuming a time-harmonic dependence ($e^{j\omega t}$) for both electric and magnetic fields, and using $\nabla \times \vec{\mathrm{H}} = \frac{\partial \vec{\mathrm{E}}}{\partial t}$ in the insulator region (where there are no free charges, hence no currents), implies $\frac{\partial H_x}{\partial y} = -j\omega\varepsilon_i E_z$ and $\frac{\partial H_x}{\partial z} = j\omega\varepsilon_i E_y$:

$$E_z = \frac{-2B\beta}{\omega\varepsilon_i}\cosh(\alpha_2 z)e^{j\beta y}, \tag{S4}$$

$$E_y = \frac{-2B\alpha_2}{j\omega\varepsilon_i}\sinh(\alpha_2 z)\, e^{j\beta y}. \tag{S5}$$

As mentioned earlier in this section, the propagating plasmonic mode along a MIM waveguide is transverse magnetic (TM) in nature, and as a result it cannot be described or analyzed using the conventional transmission line theory. However, one can use the following approximative approach to only obtain the basic geometrical parameters of the antenna and the feed.

In the near-infrared (NIR) regime, the absolute value of the permittivity of metals is very large compared to the permittivity of dielectrics like silicon dioxide (SiO_2), so that the ratio of the transverse to longitudinal component of the electric field can be written as $|E_z/E_y| = |\beta/\alpha_2||\coth(\alpha_2 z)|$, and considering ε_m and ε_d as the permitivitties of the metallic and dielectric layers, respectively, can be simplified to $|E_z/E_y| = |\surd(\varepsilon_m/\varepsilon_d\ )||\coth(\alpha_2 z)|$ which is a function that has its maximum in the middle of the insulator region. For instance, using silver (ε_m=-130.74 + j3.28 at λ_0 = 1550 nm) [5] and SiO_2 (ε_d = 2.33 at λ_0 = 1550 nm), one can immediately realize that the ratio $\left|\sqrt{\frac{\varepsilon_m}{\varepsilon_d}}\right| \approx 7.5$ and its multiplication to the diverging (at z = 0 nm) $|\coth(\alpha_2 z)|$, results in a transverse electric field component that is much larger than its longitudinal component (see

Supplementary section S3, Figure S3(a) for the present MIM geometry), where simulation results show that the transverse component of the electric field is approximately 11 times larger than the longitudinal component). Consequently, the propagating TM mode can be approximated as a TEM mode (with a small TM component), and transmission line model can be used for the basic design and determining the essential geometric parameters of the plasmonic waveguide and the patch [6], [7]. It is crucial to recognize that the derived formulas do not constitute a precise methodology for designing patch antennas in the optical regime. Their applicability is limited to specific geometries and is heavily affected by factors such as the gap between metallic plates.

Having determined E_z and H_x one can calculate the characteristic impedance (Z) of the even mode using:

$$Z = \frac{\int E_z . dz}{\oint H_x . dl}, \tag{S6}$$

where, l is the closed patch encircling the conductor. Since the magnetic field has no z-component, hence the closed path l only includes x-components from 0 to W (width of the conductor), as a result:

$$Z = -\frac{2\beta}{\alpha_2}\frac{1}{\omega\varepsilon W}\tanh\left(\alpha_2\frac{h_d}{2}\right). \tag{S7}$$

Having established the approximate preliminary relationships for the inductances, capacitances, and impedances, and using equation (S7), the basic optimal parameters of the patch can be chosen using this approximative method.

S3 Alternative Approach to Model the MIM Structures

Applying an electric field to a conductive structure, enforces its electrons to accelerate, however electrons will not reach their final velocity instantaneously, as they need to accelerate gradually.

This resistance to motion due to the electron mass, combined with the fact that in plasmonic regime the inertia of the electron gas cannot be neglected (as surface plasmons are typically associated with structures that have very small feature sizes on the order of the wavelength of visible light or smaller), is the origin of kinetic inductance. This type of inductance is obvious in Drude's complex conductivity formula [8]:

$$\sigma_m(\omega) = \frac{nq^2\tau}{m(1+\omega^2\tau^2)} - j\frac{nq^2\omega\tau^2}{m(1+\omega^2\tau^2)}, \tag{S8}$$

where, n is the number of conduction electrons, q is the charge of and electron, τ is the collision time, m is the effective mass of an electron in the metal, and ω is the angular frequency. Although kinetic inductance can be neglected at frequencies less than 100 - 110 GHz since metals typically have collision times on the order of 10^{-14} s, multiplication of ω into the collision time leads to a very small value. However, in the optical regime where frequencies are on the orders of hundreds of THz ($\approx 10^{14}$ 1/s), $\omega\tau$ is not small anymore and the imaginary part of the conductivity cannot be neglected. For the parallel plate geometry (MIM) one must consider the kinetic inductance L_k, intra-plate Faraday inductance L_{f_i}, and cross-plate Faraday inductance L_{f_c} [8] (it is widely known that kinetic inductance dominates over all other kind of inductances at large wavevectors, however in the NIR regime, all inductances mentioned above must be considered). The present analysis holds only when the plate spacing is not larger than the modal wavelength, otherwise, only a fraction of the electric field lines reaches from one plate to another, and the remaining field lines will only contribute to intra-plate capacitance. The L_K, L_{f_i}, and L_{f_c} can be obtained through the following equations [8]:

$$L_k = \frac{2}{\omega^2\delta_m W\varepsilon_0(1-\varepsilon_m)}, \tag{S9}$$

$$L_{f_i} = \frac{\mu_0}{\beta W}, \tag{S10}$$

$$L_{f_c} = \frac{\mu_0 h_d e^{\beta h_d}}{W}, \tag{S11}$$

where, $\delta_m = \left(\beta^2 - \left(\frac{\omega^2}{c^2}\right)\right)^{-\frac{1}{2}}$ is the surface wave skin depth, W is the width of the MIM structure, ε_0 and ε_m are the vacuum and metal partitivities, respectively, μ_0 is the vacuum permeability, and h_d is the distance between the two metallic plates. The total inductance L_t, which is L_k in series with the parallel equivalent of L_{f_i} and L_{f_c} (the current in the metallic plates must flow either in the plate or cross-plate) is:

$$L_t = \frac{1}{W}\left[\frac{\mu_0 h_d}{\beta h_d + e^{-\beta h_d}} + \frac{2}{\omega^2 \delta_m \varepsilon_0 (1-\varepsilon_m)}\right]. \tag{S12}$$

With all of the parameters at hand and considering that the total capacitance (intra- and cross-plate capacitance) $C_t = \varepsilon_0(\beta d + e^{-k h_d})\frac{W}{h_d}$ [8], the impedance can be obtained using $Z = \sqrt{\frac{L_t}{C_t}}$.

S4 Obtaining Widths of the Patches

As has been mentioned in the main manuscript, in the near-infrared (NIR) regime, the absolute value of the permittivity of metals is very large in comparison with the permittivity of dielectrics like silicon dioxide (SiO_2), so that the ratio of the transverse to longitudinal component of the electric field can be approximated as $\left|\sqrt{\frac{\varepsilon_m}{\varepsilon_d}}\right|$ [6], where ε_m is the metal permittivity and ε_d is permittivity of the dielectric layer sandwiched between two metallic layers. For instance, as shown in the main manuscript, using silver (ε_m=-130.74 + j3.28 at λ_0 = 1550 nm) [5] and SiO_2 (ε_d = 2.33 at λ_0 = 1550 nm), one can immediately realize that the transverse component of the electric field is approximately 7.5 times larger than its longitudinal component. It should be noted the ratio $\left|\sqrt{\frac{\varepsilon_m}{\varepsilon_d}}\right|$ has been derived from an approximate equation of the wavevector in MIM waveguides, the

ratio of the transverse to longitudinal components of the electric field in the proposed MIM waveguide is typically larger (on the order of 10 to 13). This is clearly shown in Figure S3(a), where the magnitude of the *z*- (transverse) and *y*- (longitudinal) components of the electric field are plotted versus *z*-direction. Consequently, the propagating TM mode can be approximated as a TEM mode (with a small TM component), and the transmission line model can be used for basic design and determining the essential geometric parameters of the plasmonic waveguide and the patch. However, it is crucial to recognize that this approach represents a mere approximation. The derived formulas by no means constitute a comprehensive or precise methodology for designing patch antennas in the optical regime and their applicability is constrained to specific scenarios, heavily influenced by factors such as the gap between metallic plates.

On the other hand, the effective index of the mode for the present geometry can be approximately calculated using the following equation [9]:

$$n_{eff} = \sqrt{\varepsilon_d}\left(\sqrt{1 + \frac{\lambda_0}{\pi h_d \sqrt{-\varepsilon_m}}\sqrt{1 + \frac{\varepsilon_d}{-\varepsilon_m}}}\right), \tag{S13}$$

where ε_d is the permittivity of the dielectric layer, ε_m is the permittivity of the metallic layers, λ_0 is the free space wavelength, and h_d is the height of the dielectric layer in the MIM structure (h_d = 20 nm for all of the antennas in this work). The obtained values for the effective index of the mode are plotted in Figure S3(b). Consequently, width of a patch is obtained from the following equation [10]:

$$W_p = \frac{c}{2f_r}\sqrt{\frac{2}{{n_{eff}}^2 + 1}}, \tag{S14}$$

where c and f_r are the free space velocity of light and resonant frequency of the patch, respectively.

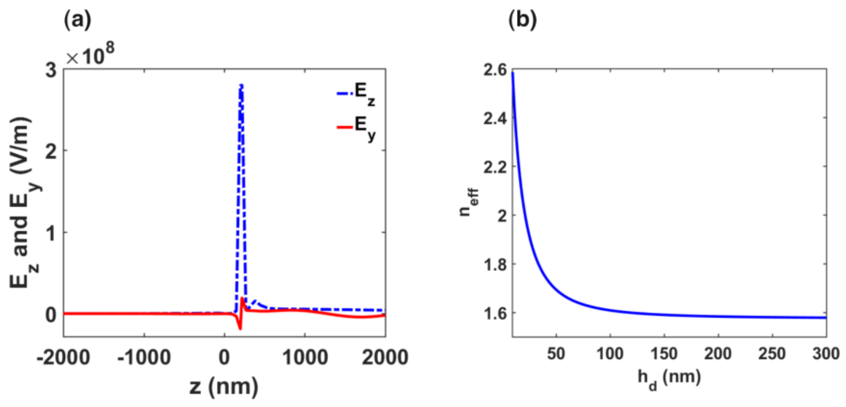

Figure S3. Magnitude of the transverse and longitudinal components of the electric field plotted versus a 1D cut along the *z*-axis, (b) effective index of the propagating mode inside the MIM waveguide with a width (W_g) = 100 nm.

S5 Proposed Architecture and Hyperparameter Optimization

We have utilized Optuna [11], a hyperparameter optimization framework, to select the optimal architecture and hyperparameters for our neural network-based surrogate solver. Optuna provides various samplers and pruners to explore and optimize hyperparameters. It also features a web-based interface and easy-to-use visualization tools. In this work, we have utilized a pure random sampler to thoroughly explore the hyperparameter space and a median pruner to speed up the entire process.

We have performed the hyperparameter optimization for 300 trials with the following parameter distributions: the batch size has a categorical distribution with the values of (64, 128, 256, 512), learning rate has a logarithmic uniform distribution with a range from $1e^{-5}$ to $1e^{-1}$, regularization weight (only for fully-connected layers) has a logarithmic uniform distribution with a range from $1e^{-10}$ to $1e^{-6}$, the configuration of fully-connected layers has a categorical distribution with values described in Table S1, the configuration of convolutional layers has a

categorical distribution with values described in Table S2, and the activation of convolutional layers has a categorical distribution with true and false values.

Table S3. shows the top five trials and their corresponding configuration and test set error. We have selected the trial (#65) for the rest of the experiments in our work.

The selected architecture has five fully connected layers, with each layer consisting of 512 neurons that use a leaky relu as the activation function. The last fully connected layer is followed by three convolutional blocks that estimate S_{11} and the radiation pattern at $\varphi = 0^\circ$ and $\varphi = 90^\circ$.

Table S1. Configuration of fully connected layers.

**Index**	**Number of layers and neurons**	**Total number of trainable weights**
1	128×256	32,768
2	128×256×512	163,840
3	512×512	262,144
4	512×512×512	524,288
5	128×256×512×1024	688,128
6	512×512×512×512	786,432
7	512×512×512×512×512	1,048,576
8	128×256×512×1024×1024	1,736,704

Table S2. Configuration of convolutional layers.

**Index**	**Input dimension**	**Number of layers and channels**	**Number of trainable weights**
1	6×64	64, 32, 16, 8	20,376
2	6×64	64, 64, 32, 32	33,888
3	6×128	128, 64, 32, 16	81,456
4	6×128	128, 128, 64, 64	135,360

Table S3. Configuration of top five trials.

Trial number	Batch size	Learning rate (e^{-4})	Regularization weight (e^{-8})	Configuration of FC layers	Configuration of Conv layers	Enable convolutional layers	S_{11} test set error	Overall test set error
65	256	9.27	0.02	7	2	True	0.53	1.29
99	256	9.82	0.01	7	3	True	0.55	1.31
112	64	1.16	0.37	6	3	True	0.57	1.44
196	256	4.60	3.75	7	1	True	0.59	1.45
265	64	13.63	1.70	5	1	True	0.55	1.36

Each convolutional block consists of four layers. Each layer includes an up-sampling function, followed by a convolutional and a batch normalization layer. A leaky relu is used as an activation function at the end of each layer. The kernel size used is 3×1, and the number of channels in each layer is as follows: 64, 64, 32, 32.

Optuna also calculates the importance of each hyperparameter using the fANOVA algorithm [12]. Figure S4 illustrates the importance of our hyperparameters. As the results show, having convolutional layers after the fully connected layers significantly improves the network's accuracy.

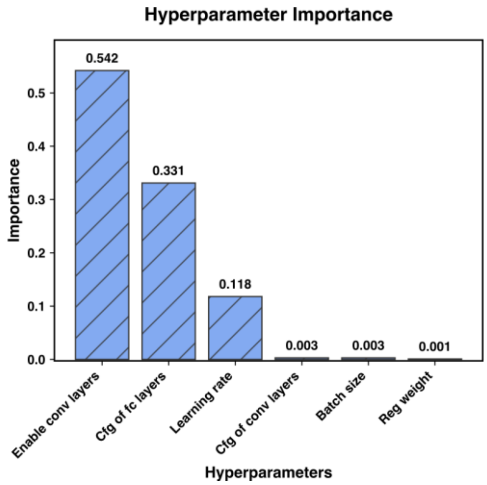

Figure S4. Hyperparameter importance diagram.

The number of fully connected layers along with the learning rate are significant factors in the learning process as well. On the other hand, the configuration of convolutional layers, the batch size, and the regularization weight had no significant contribution to the overall accuracy.

S6 Extended Results

This section demonstrates the extended results achieved by training the proposed network on the dataset with three and four parameters. Figure S5 depicts the prediction accuracy and error distribution of the proposed surrogate solver for the device with four degrees of freedom (dataset with four parameters). The qualitative results of the inverse design verification experiment (Section 2.4.1 of main manuscript) for the device with four degrees of freedom are illustrated in Figure S6.

The error distribution of the verification experiments (Section 2.4.1 of main manuscript) for the device with three and four degrees of freedom are shown in Figure S7 and Figure S8, respectively.

Figure S9-S12 demonstrate the extended query-based results of Section 3 of the main manuscript.

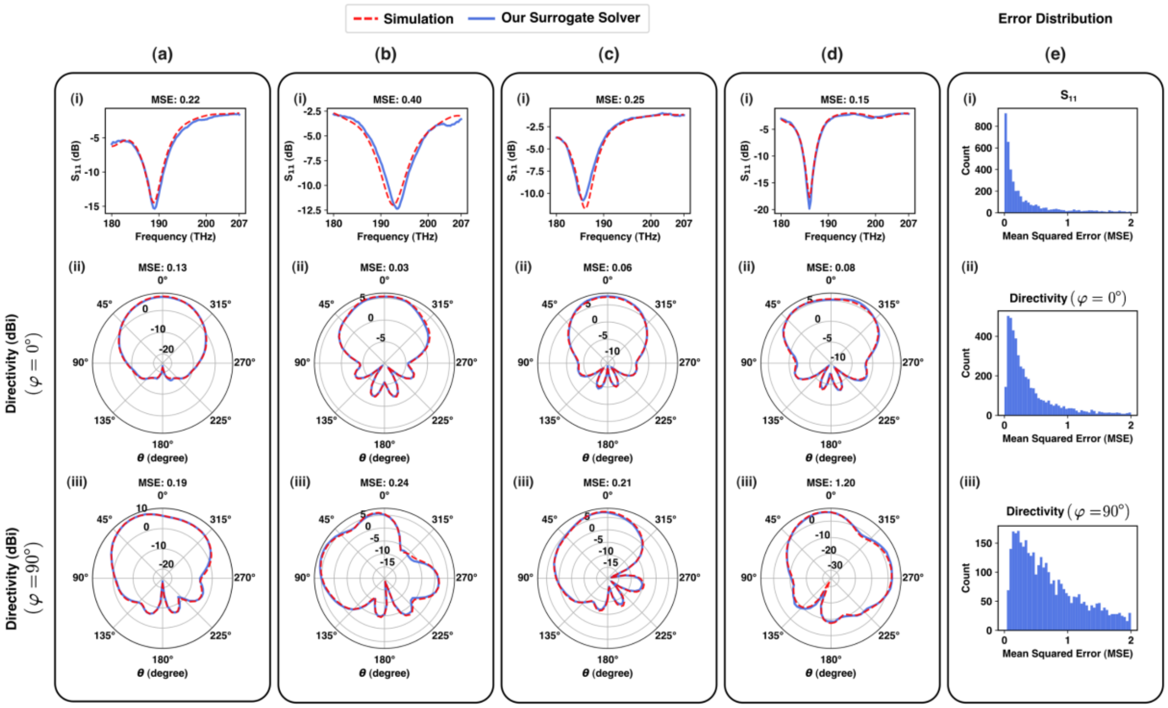

Figure S5. Prediction accuracy and the error distribution of the proposed surrogate solver trained on the dataset of four parameters. (a-d) the simulated response and the predicted response of four devices, (e) the error distribution of each response type.

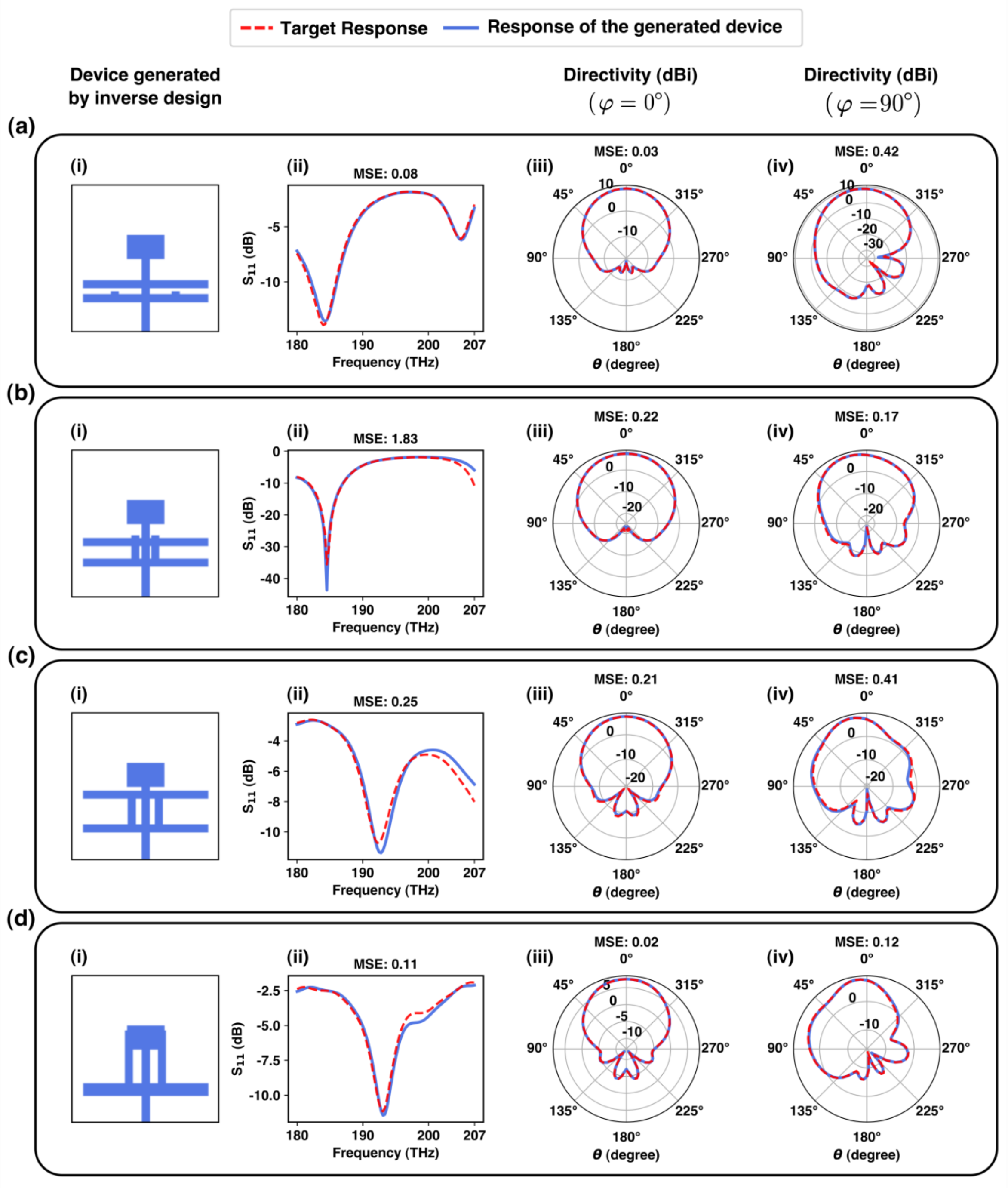

Figure S6. Inverse design verification experiment with the goal of generating single optimal devices given the target responses (device with four degrees of freedom).

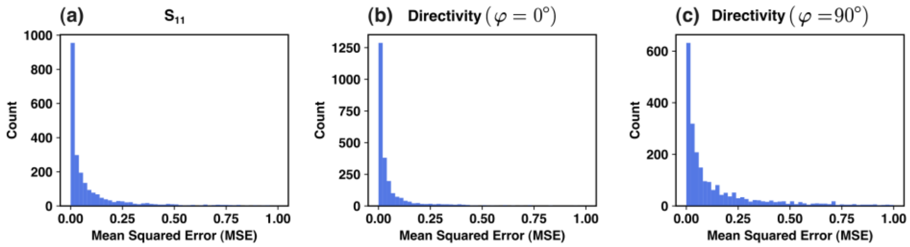

Figure S7. The error distribution of inverse design verification experiment for the device with three degrees of freedom.

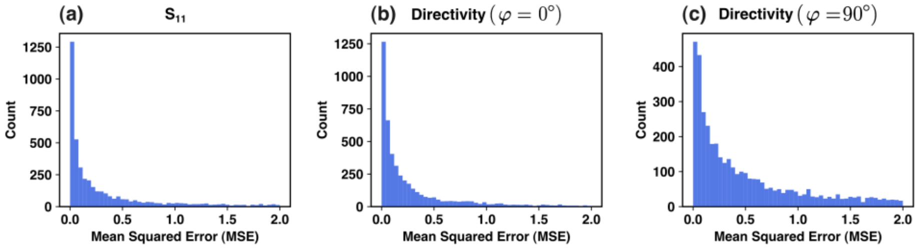

Figure S8. The error distribution of inverse design verification experiment for the device with four degrees of freedom.

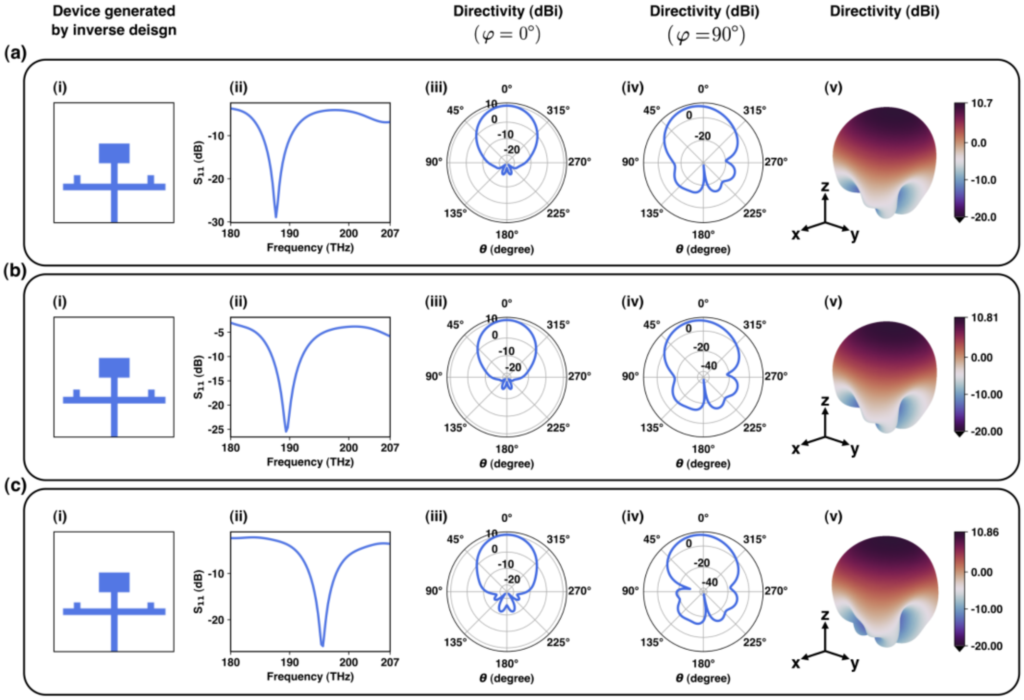

Figure S9. (a-c) Exemplary single band nanoantennas generated by the inverse design framework for f = 188.5 THz, f = 190 THz, f = 195 THz with S_{11} < -10 dB and the highest possible directivity in $\varphi = 0^\circ$ and $\varphi = 90^\circ$ planes. Each subfigure (i-v) in panels (a-c) shows the schematic of the device, S_{11}, directivity in $\varphi = 0^\circ$ plane, directivity in $\varphi = 90^\circ$ plane, and the 3D radiation pattern for each of the devices, respectively.

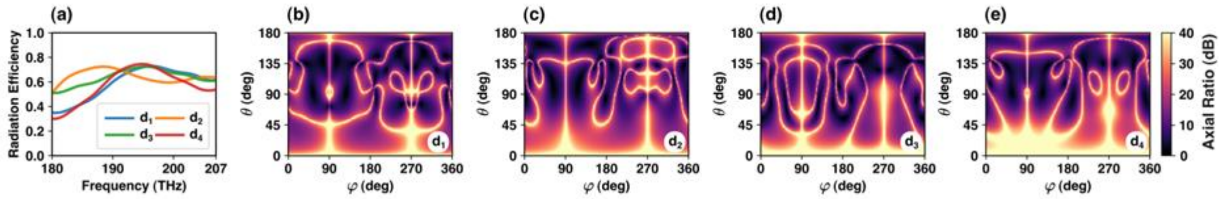

Figure S10: Radiation efficiency and axial ratio of the single band nanoantennas designed by the proposed inverse design framework. (a) Radiation efficiency of devices d_1-d_4 shown in Figure 7 of the main manuscript, (b-e) axial ratio of devices d_1-d_4, respectively.

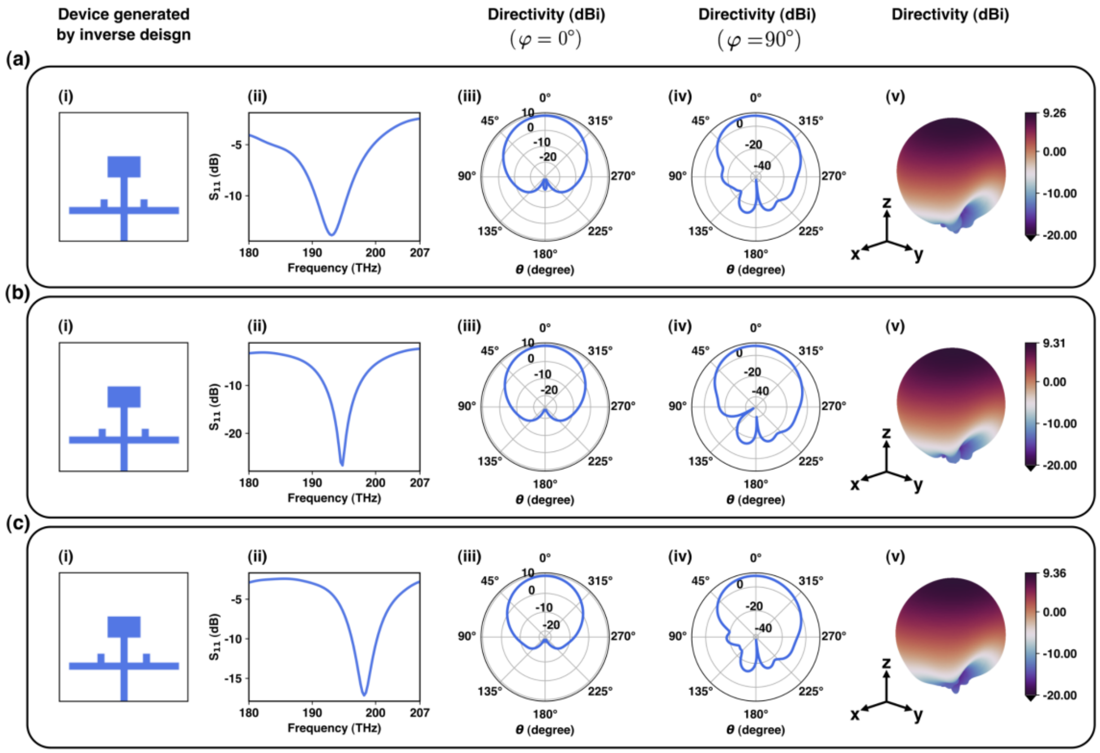

Figure S11. (a-c) Exemplary backlobe-suppressed single band nanoantennas generated by the inverse design framework for f = 193.5 THz, f = 195 THz, f = 198.5 THz with S_{11} < -10 dB and the highest possible directivity in $\varphi = 0^{\circ}$ and $\varphi = 90^{\circ}$ planes, and a suppressed radiation in $\theta = 180°$. Each subfigure (i-v) in panels (a-c) shows the schematic of the device, S_{11}, directivity in $\varphi = 0^{\circ}$ plane, directivity in $\varphi = 90^{\circ}$ plane, and the 3D radiation pattern for each of the devices, respectively.

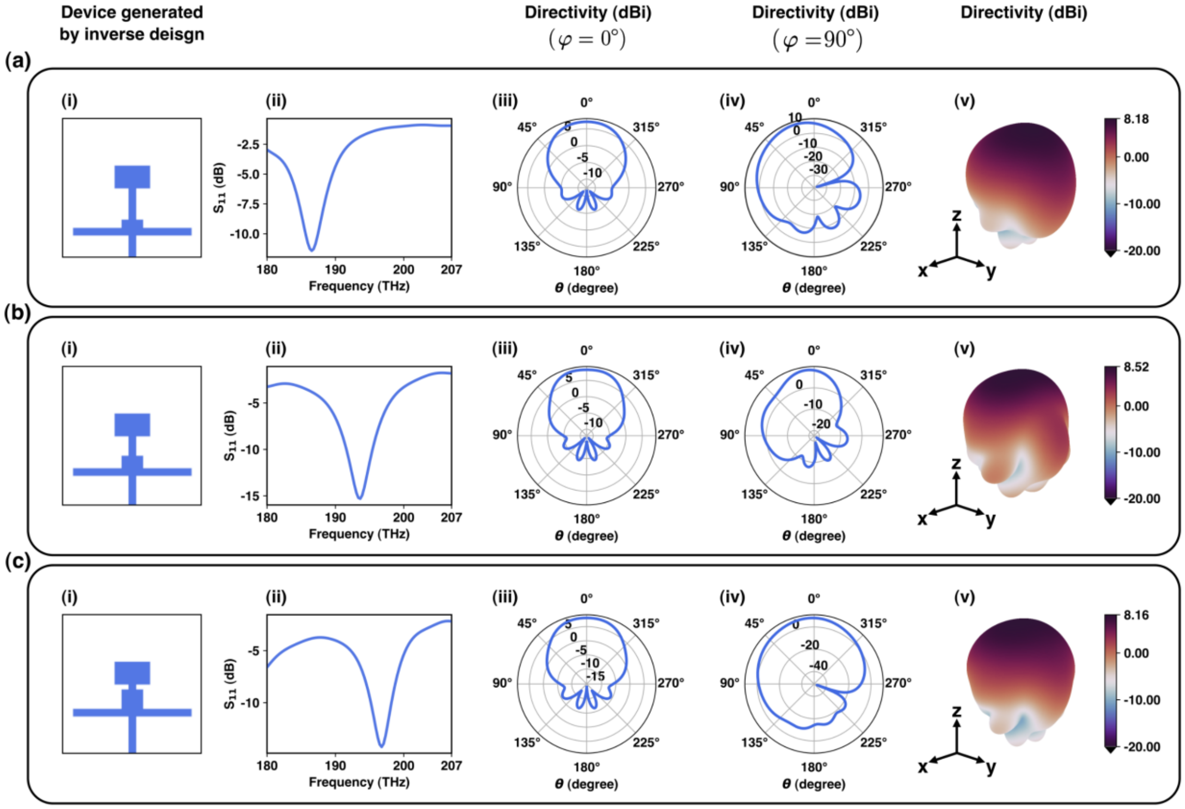

Figure S12. (a-c) Exemplary constraint-imposed single band nanoantennas generated by the inverse design framework, where the location of the arm of the T-stub is fixed at 50 nm and the network is tasked to generate devices with S_{11} < -10 dB and directivity > 8 dBi in $\varphi = 0^\circ$ and $\varphi = 90^\circ$ planes, for f = 186.5 THz, f = 193.5 THz, f = 196.5 THz. Each subfigure (i-v) in panels (a-c) shows the schematic of the device, S_{11}, directivity in $\varphi = 0^\circ$ plane, directivity in $\varphi = 90^\circ$ plane, and the 3D radiation pattern for each of the devices, respectively.

S7 Nonlinearity of the problem

Following a through discussion on nonlinearity of the current problem despite having 3 and 4 parameters for each case, it would be beneficial to show some instances of this nonlinearity. Figure S13 shows two examples of this nonlinearity, where a slight change in the location of the arm of the T-stub (D_a) by steps of 20 nm, in two different nanoantennas with two sets of fixed parameters (D_{st1}, L_a) and (D_{st1}', L_a'), leads to nonlinear changes in the S_{11}. The step size of 20 nm was chosen because the average distance between two closest points is about 20 nm. Assuming that there are 36.8 points in each dimension and the average range of motion of the parameters is 700 nm, this means that there is a 20 nm distance between each configuration.

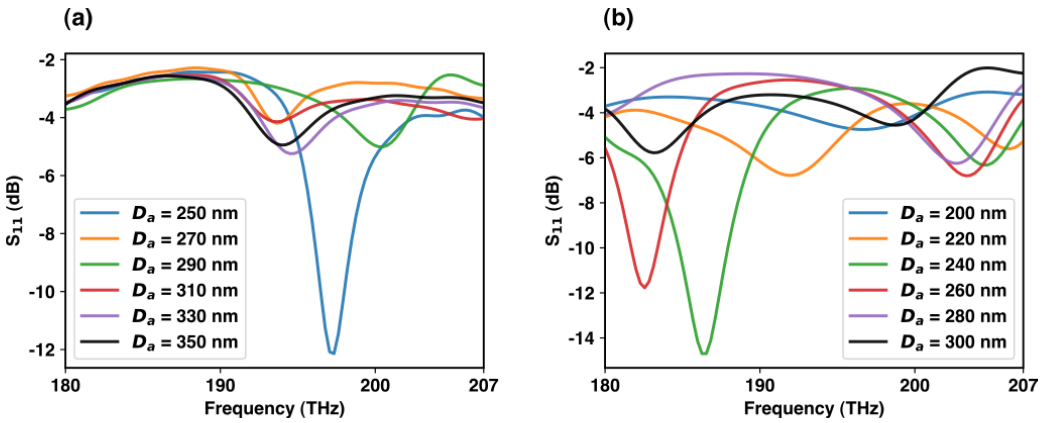

Figure S13. Illustration of the non-linearity of the problem, (a) and (b) S_{11} of two different antennas with two sets of fixed parameters (D_{st1}, L_a) and (D_{st1}', L_a') for the first and the second antennas while D_a is changed with steps of 20 nm, showing large variations in S_{11} of each antenna with a slight change in just one parameter (D_a). The step size of 20 nm was chosen because the average distance between two closest points is about 20 nm. Assuming that there are 36.8 points in each dimension and the average range of motion of the parameters is 700 nm, this means that there is a 20 nm distance between each configuration.

S8 Comparison to lookup table algorithm

The mapping between the devices and their responses is highly complex and non-linear. As a result, many devices with desirable responses are not included in the training set. However, the multi-head convolutional neural network has the capability to learn these mappings and apply them to new, unseen regions. Its performance differs from that of a simple lookup table. Figure S14 shows several examples where the surrogate solver successfully generates useful devices that were not part of the training set.

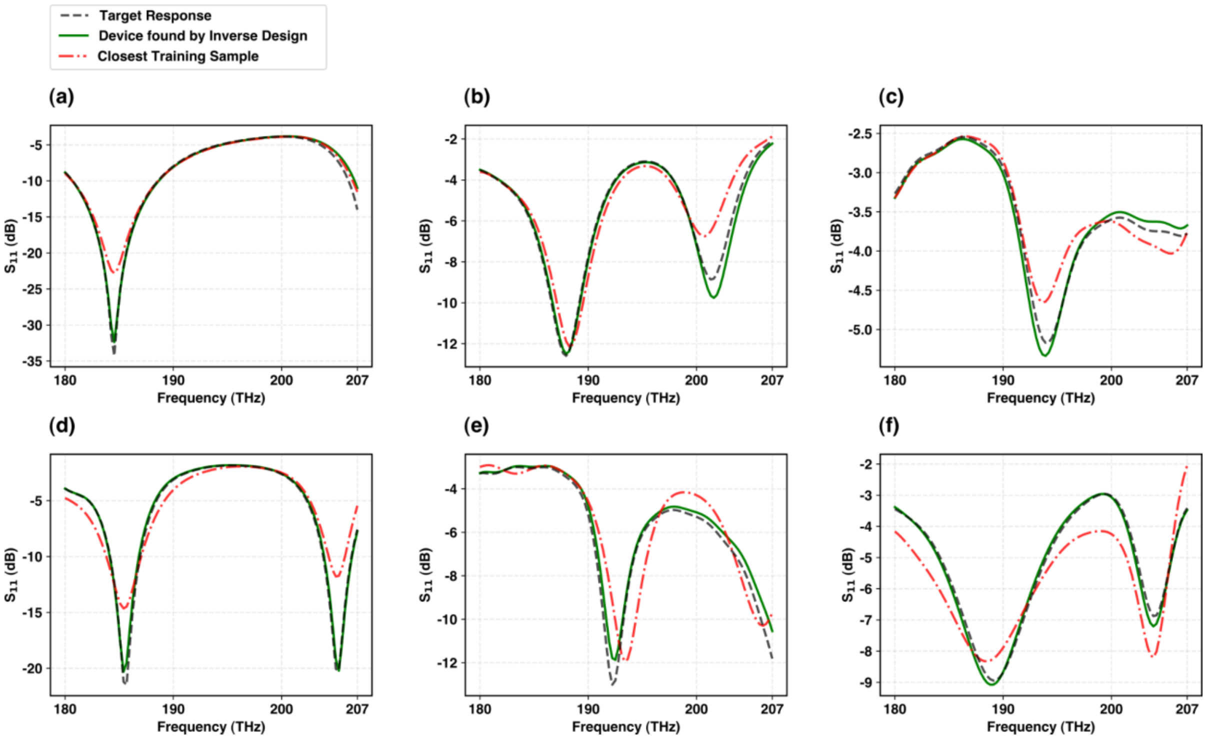

Figure S14: Qualitative examples showing that the surrogate solver behavior is different than a lookup algorithm. The surrogate solver has the ability to generalize to unseen data. (a-f) several instances where the surrogate solver has found a more accurate device given the target response comparing to those samples exists in the training set. The blacked dashed line shows the target response that the device must have, the green line shows the response of the device found by our inverse design framework, and the red dashed line shows the response of the closest training sample to the target response.

References

[1] M. D. McKay, R. J. Beckman, and W. J. Conover, “A Comparison of Three Methods for Selecting Values of Input Variables in the Analysis of Output from a Computer Code,” *Technometrics*, vol. 21, no. 2, p. 239, May 1979, doi: 10.2307/1268522.

[2] R. L. Iman, J. C. Helton, and J. E. Campbell, “An Approach to Sensitivity Analysis of Computer Models: Part I—Introduction, Input Variable Selection and Preliminary Variable Assessment,” *Journal of Quality Technology*, vol. 13, no. 3, pp. 174–183, Jul. 1981, doi: 10.1080/00224065.1981.11978748.

[3] J. C. Helton and F. J. Davis, “Latin hypercube sampling and the propagation of uncertainty in analyses of complex systems,” *Reliability Engineering & System Safety*, vol. 81, no. 1, pp. 23–69, Jul. 2003, doi: 10.1016/S0951-8320(03)00058-9.

[4] S. A. Maier, *Plasmonics: Fundamentals and Applications*. New York, NY: Springer US, 2007. doi: 10.1007/0-387-37825-1.

[5] P. B. Johnson and R. W. Christy, “Optical Constants of the Noble Metals,” *Phys. Rev. B*, vol. 6, no. 12, pp. 4370–4379, Dec. 1972, doi: 10.1103/PhysRevB.6.4370.

[6] L. Yousefi and A. C. Foster, “Waveguide-fed optical hybrid plasmonic patch nano-antenna,” *Opt. Express*, vol. 20, no. 16, p. 18326, Jul. 2012, doi: 10.1364/OE.20.018326.

[7] B. A. Nia, L. Yousefi, and M. Shahabadi, “Integrated Optical-Phased Array Nanoantenna System Using a Plasmonic Rotman Lens,” *J. Lightwave Technol.*, vol. 34, no. 9, pp. 2118–2126, May 2016, doi: 10.1109/JLT.2016.2520881.

[8] M. Staffaroni, J. Conway, S. Vedantam, J. Tang, and E. Yablonovitch, “Circuit analysis in metal-optics,” *Photonics and Nanostructures - Fundamentals and Applications*, vol. 10, no. 1, pp. 166–176, Jan. 2012, doi: 10.1016/j.photonics.2011.12.002.

[9] S. Collin, F. Pardo, and J.-L. Pelouard, “Waveguiding in nanoscale metallic apertures,” *Opt. Express*, vol. 15, no. 7, p. 4310, 2007, doi: 10.1364/OE.15.004310.

[10] C. A. Balanis, *Antenna theory: analysis and design*, Fourth edition. Hoboken, New Jersey: Wiley, 2016.

[11] T. Akiba, S. Sano, T. Yanase, T. Ohta, and M. Koyama, “Optuna: A Next-generation Hyperparameter Optimization Framework,” in *Proceedings of the 25th ACM SIGKDD International Conference on Knowledge Discovery & Data Mining*, Anchorage AK USA: ACM, Jul. 2019, pp. 2623–2631. doi: 10.1145/3292500.3330701.

[12] F. Hutter, H. Hoos, and K. Leyton-Brown, “An Efficient Approach for Assessing Hyperparameter Importance,” in *Proceedings of the 31st International Conference on Machine Learning*, E. P. Xing and T. Jebara, Eds., in Proceedings of Machine Learning Research, vol. 32. Bejing, China: PMLR, Jun. 2014, pp. 754–762. [Online]. Available: https://proceedings.mlr.press/v32/hutter14.html
